# Feeding for Well-Being: Porcine Blood Hydrolysate Supplementation Improves Metabolic and Welfare-Related Traits in Farmed Gilthead Sea Bream (*Sparus aurata*)

**DOI:** 10.3390/ijms262110725

**Published:** 2025-11-04

**Authors:** Cristina Moreno-Mariscal, Paul Holhorea, Federico Moroni, Leticia Mora, Fidel Toldrá, Jaume Pérez-Sánchez

**Affiliations:** 1 Instituto de Agroquímica y Tecnología de Alimentos (IATA-CSIC), Avenue Agustín Escardino 7, 46980 Paterna, Spain; cmormar@iata.csic.es (C.M.-M.); ftoldra@iata.csic.es (F.T.); 2 Instituto de Acuicultura Torre de la Sal (IATS-CSIC), 12595 Ribera de Cabanes, Spain; paul.holhorea@csic.es (P.H.); jaime.perez.sanchez@csic.es (J.P.-S.)

**Keywords:** enzymatic hydrolysis, functional feeds, stress metabolic markers, aggressive behavior, swimming performance, basal metabolism

## Abstract

The revalorization of animal by-products, such as porcine blood, is a key strategy for sustainable aquaculture and circular economy practices. This study aimed to fill the existing knowledge gap on the effects of spray-dried porcine blood hydrolysate (PBSH), assessing its potential as a functional feed ingredient for gilthead sea bream. Two practical diets were formulated: a control diet containing 5% blood meal, and a PBSH diet including 5% PBSH previously characterized in vitro. The results indicated that the PBSH diet promoted lower hepatosomatic index, a down-regulation of key hepatic lipogenic enzymes (*scd1b*, *hl*, *lpl*), and a better stress condition with lower circulating levels of glucose and cortisol and a reduction in aggressive attacks. Positive findings were also achieved in energy management, obtaining lower metabolic rates along with an enhanced swimming performance (20% increase in the critical speed) and a quicker weigh recovery after a fasting period. The PBSH diet also shaped the intestinal bacterial composition, determining a redistribution of abundant genera including *Aureimonas* and *Halomonas*. Ultimately, this study demonstrated that PBSH would act as a functional ingredient capable of enhancing fish energy management and resilience in the face of stressful events, exhibiting a transient transcriptional modulation, yet persistent physiological and welfare benefits.

## 1. Introduction

The aquaculture sector has shown exponential growth in recent decades, consolidating its position as one of the main sources of animal protein production globally. By 2022, total aquatic animal production reached 185 million tons (live weight), with an annual growth rate of 3.2%. Projections indicate that this trend could increase to 205 million tons by 2032 [1]. However, due to the limited availability of fishmeal and fish oil (FM; FO) and their environmental impact, the sector’s future development mainly depends on the use of more sustainable raw materials and ingredients that do not compromise animal health and performance [2].

In this context, one of the principal alternatives, which guarantees a good conversion ratio and an adequate nutritional profile, is animal by-products (ABPs). These resources include insect proteins, microbial biomass, and processed animal proteins (PAP), derived from the valorization of meat industry by-products such as poultry meat meal, feather meal, whole dried blood, and plasma proteins [3]. These by-products, and in particular porcine blood, are especially interesting thanks to their elevated content of protein with high biological value (around 18%), low cost, and wide availability, resulting from the large quantities generated daily in slaughterhouses [4,5]. Hence, their reutilization does not only enhance the sustainability and efficiency of the aquaculture sector but also promote a circular economy within food industry by reducing waste and adding value to these resources [6,7]. The most common process for the further revalorization of such ABPs is represented by enzymatic hydrolysis [8,9,10,11]. This procedure, in fact, has proven to be an effective and sustainable method that allows for obtaining extracts rich in bioactive peptides [12,13] and free amino acids (FAAs), which can play a beneficial role in the fish’s organism, improving protein digestibility and bioaccessibility, and thus contribute to their well-being [14]. This nutritional versatility makes protein hydrolysates an excellent potential functional ingredient for animal feeds. However, despite its large application in terrestrial species, in aquaculture, the inclusion of this beneficial protein source in diets is still not widespread, and its effects on farmed organisms’ physiology are far from being deeply investigated, especially when considering porcine blood, which represents an ingredient commonly used in fish feed formulations.

Accordingly, to fill this knowledge gap and enhance the valorization of an underutilized by-product, the aim of this study was to evaluate the biological activity of PBSH through its incorporation as a functional ingredient in fish feed. The test feeding trial was performed in gilthead sea bream (*Sparus aurata*, Linnaeus, 1758), a highly representative marine farmed fish in European aquaculture [15]. The study assessed the growth performance and welfare status in fast-growing juvenile fish using a multi-layer metabolic, transcriptional, and gut microbiota approach, including measurements of behavior, swimming performance, dynamics of fasting weight loss, and weight regain during the refeeding phase, also evaluating the temporal effects of PBSH feed supplementation at both short- and medium-term scales.

## 2. Results

### 2.1. Growth Performance and Feeding Behavior

During the 10-week trial, all fish exhibited optimal growth regardless of diet, registering a specific growth rate (SGR) of 2.29–2.3% and a feed conversion ratio (FCR) of 1.0–1.01 at the end of the trial (Table 1). Regarding organosomatic indexes, fish that were fed the PBSH diet highlighted a significant smaller hepatosomatic index (HSI), although a significant decrease in liver weight was not found. Significant differences were also detected in pre-feeding behavior during the 30 min before the daily feed administration, which demonstrated a significant 33% reduction in the frequency of attacks between individuals of fish fed the PBSH diet in comparison to the control group (CTRL).

### 2.2. Histopathology Evaluation and Blood Biochemistry

**Figure 1:**
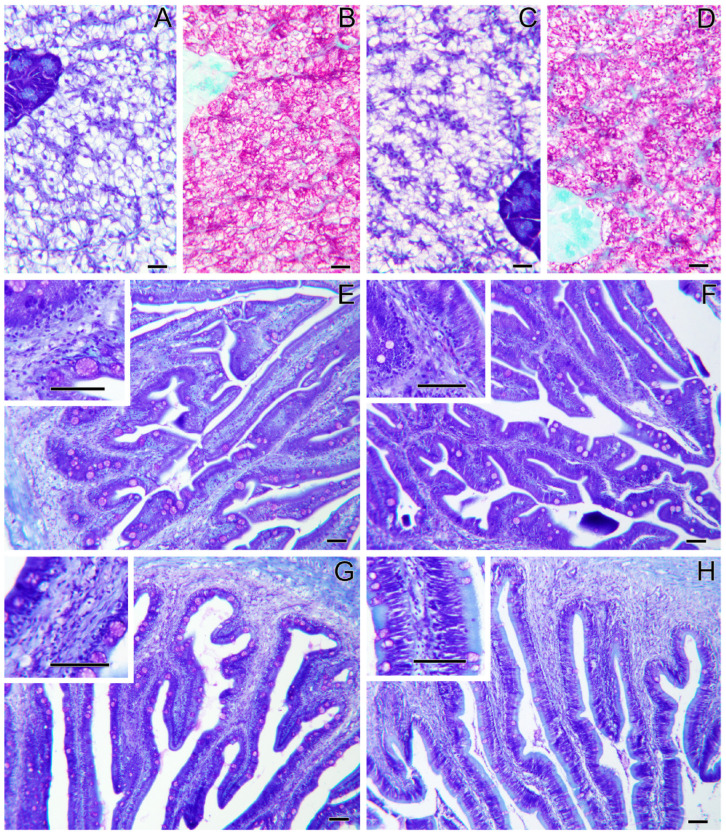
Liver and intestine histology of gilthead sea bream fed the CTRL diet (**A**,**B**,**E**,**G**), and the PBSH diet (**C**,**D**,**F**,**H**). Liver sections (**A**–**D**) present no histopathological signs, and no differences are observed among groups. The anterior (**E**,**F**) and the posterior (**G**,**H**) intestinal segments also present no histopathological signs and no differences among groups. Giemsa staining (**A**,**C**,**E**–**H**) and PAS staining (**B**,**D**). Scale bars = 20 µm in liver sections and = 50 µm in intestinal sections.

**Figure 2:**
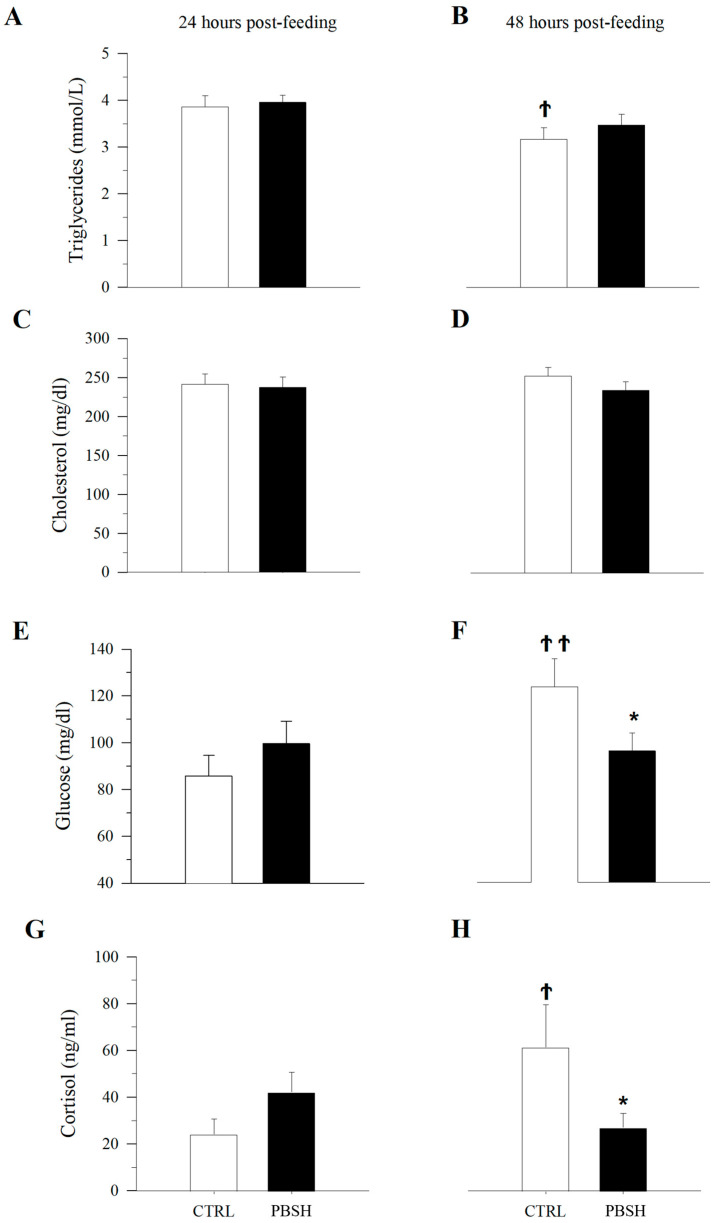
Effect of diet on blood biochemistry. Plasma levels of triglycerides (**A**,**B**), cholesterol (**C**,**D**), glucose (**E**,**F**), and cortisol (**G**,**H**) after 24 and 48 h post-feeding. *, indicates statistically significant differences between fish fed CTRL and PBSH diets at a given post-feeding time (Student’s *t*-test, * *p* < 0.1). Ϯ, indicates statistically significant differences at a given time for fish fed CTRL or PPH diets (Student’s *t*-test, Ϯ *p* < 0.1, ϮϮ *p* < 0.05).

Histopathological evaluation did not highlight any alteration due to nutritional intervention in both liver and intestine samples (Figure 1). No differences were observed in the degree of glycogen and lipid storage in hepatocytes (Figure 1A–D). In both intestinal segments (anterior and posterior intestine: AI, PI), inflammatory markers, goblet cell, and immune cell numbers were found at basal levels. Furthermore, no morphological differences were detected in the analyzed parameters, which included vacuolation and structure of the mucosal fold and submucosal thickness (Figure 1E,F). Hematic levels of triglycerides and cholesterol (Figure 2A–D) showed no differences between dietary groups, whereas a decreasing trend (*p* < 0.1) was recorded between the first and second sampling of triglyceride’s values. In fish fed the PBSH diet, the typical blood stress markers, glucose, and cortisol values (Figure 2E–H) registered a downward tendency compared to the control group, although not significant (*p* < 0.1), after 48 h post-feeding. In addition, both of these biochemical parameters showed an increase between the first and second sampling point in the CTRL.

### 2.3. Tissue Gene Expression Profiles

Hepatic gene expression highlighted 26 differentially expressed genes with an overall down-regulation registered in PBSH fish in comparison to the CTRL (Table S4). These nutritionally regulated genes are equally distributed between the macro functions indicated in Table 2 for the liver PCR array, including markers of GH/GF system, lipid metabolism, oxidative metabolism, and antioxidant defense, which suggests a general effect carried out by the experimental diet on the entire functional profile. This differential gene expression was mostly observed in the first sampling point (24 h post-feeding), as at 48 h post-feeding, most of the observed effects disappeared, with the exceptions of insulin-like growth factor binding protein 1b (*igfbp1b*), which maintained significantly higher values in PBSH fish, and the elongation of very long chain fatty acids 5 (*elovl5*) gene, which registered a significant up-regulation at 48 h post-feeding instead of the observed down-regulation trend at 24 h post-feeding. Examining in more detail the temporal influence on liver gene expression, the results indicated a total of 27 genes that exhibited a significant difference between 24 and 48 h post-feeding. Apart from sterol regulatory element-binding proteins 1 (*srebp1*), fatty acid desaturase 2 (*fads2*), and stearoyl-CoA desaturase 1b (*scd1b*) genes, which exhibited significantly lower values in the second sampling time, all the other genes showed a general up-regulated pattern 48 h post-feeding. Within this latter group, insulin-like growth factor-I (*igf1*), Insulin-like growth factor binding protein 2a (*igfbp2a*), cholesterol 7-alpha-monooxygenase (*cyp7a1*), hormone sensitive lipase (*hsl*), peroxisome proliferator-activated receptor α (*pparα*), fatty acid translocase/CD36 (*fat/cd36*), proliferator-activated receptor gamma coactivator 1 alpha (*pgc1α*), and sirtuins (*sirt3.1a/b*; *sirt3.2*; *sirt5b*) registered significant differences between the two sampling points with no influence of the diet. On the contrary, the rest of differentially expressed genes highlighted significant variation between sampling times due to the influence of diet in the first sampling, and the restoration of similar conditions between CTRL and PBSH groups 48 h post-feeding. Altogether, these results strongly suggest a transient effect of diet on the liver gene expression profile, which fades in the second sampling point.

The AI gene expression profile revealed a total of six genes from the total reported in Table 3 with a significant down-regulation pattern in association to the PBSH diet supplementation (Table S5). This is the case for some genes that belong in immunoregulatory response, such as transcription factor HES-1-B (*hes1b*), gap junction Cx32.2 protein (*cx32.2*), and interleukin-12 subunit beta (*il12β*), which would support some anti-inflammatory effect over time. Immunoglobulin T membrane-bound form (*igt-m*) exhibited the same trend in the 48 h post-feeding sampling time, although not strictly significant (*p* = 0.051). Conversely, an up- regulation was registered for C-C chemokine receptor type 11 (*ccr11*) in the first sampling point, while interleukin-6 and -10 (*il6*; *il10*) were up-regulated in both sampling points. Regardless of diet, the intestine results also showed a clear temporal pattern, which resulted in a trend opposite to that previously described for the liver. Such changes to gene expression profile would be indicative of the acquisition of a pro-inflammatory profile with the advancing fasting time (24–48 h post-feeding), which was driven by the up-regulation of 13 genes mostly belonging to cytokines and chemokine-related proteins, such as interleukin-15 (*il15*), interleukin-34 (*il34*), C-C chemokine receptor type 3 and 9 (*ccr3*, *ccr9*), but also T cell and monocyte/macrophage markers and pattern recognition receptors (PRRs), including cluster of differentiation 4 and 8 beta (*cd4-1*; *cd8b*), macrophage colony-stimulating factor 1 receptor 1 (*csf1r1*), macrophage mannose receptor 1 (*mrc1*), CD302 antigen (*cd302*), and fucolectin (*fcl*). With the same functional interpretation, the down-regulation of proliferating cell nuclear antigen (*pcna*) and mucin 2 (*muc2*) also highlighted an altered state of the intestine. On the contrary, *il12β* and interleukin-8 (*il8*) genes showed significant lower values after 48 h post-feeding.

**Figure 3:**
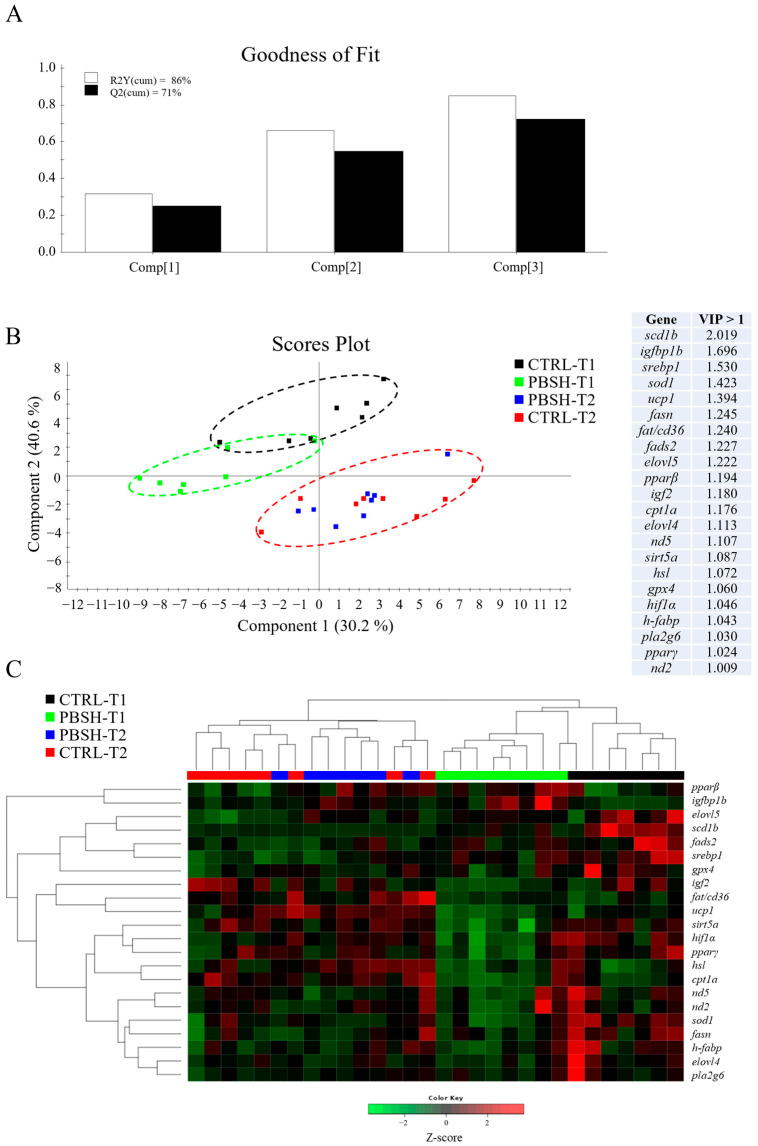
(**A**) Goodness of fit representing the cumulative explained [R2Y(cum), white bars] and predicted [Q2(cum), black bars] variance of each component. (**B**) Two-dimensional partial least-squares discriminant analysis (PLSDA) score plot of liver data constructed using the two first components in the model. Discriminant genes are ordered by variable importance in the projection (VIP). T1 and T2 refer to samples taken 24 or 48 h post-feeding. (**C**) Heatmap representing the abundance distribution (Z-score) of the genes identified to be driving the separation by diet (VIP ≥ 1).

**Figure 4:**
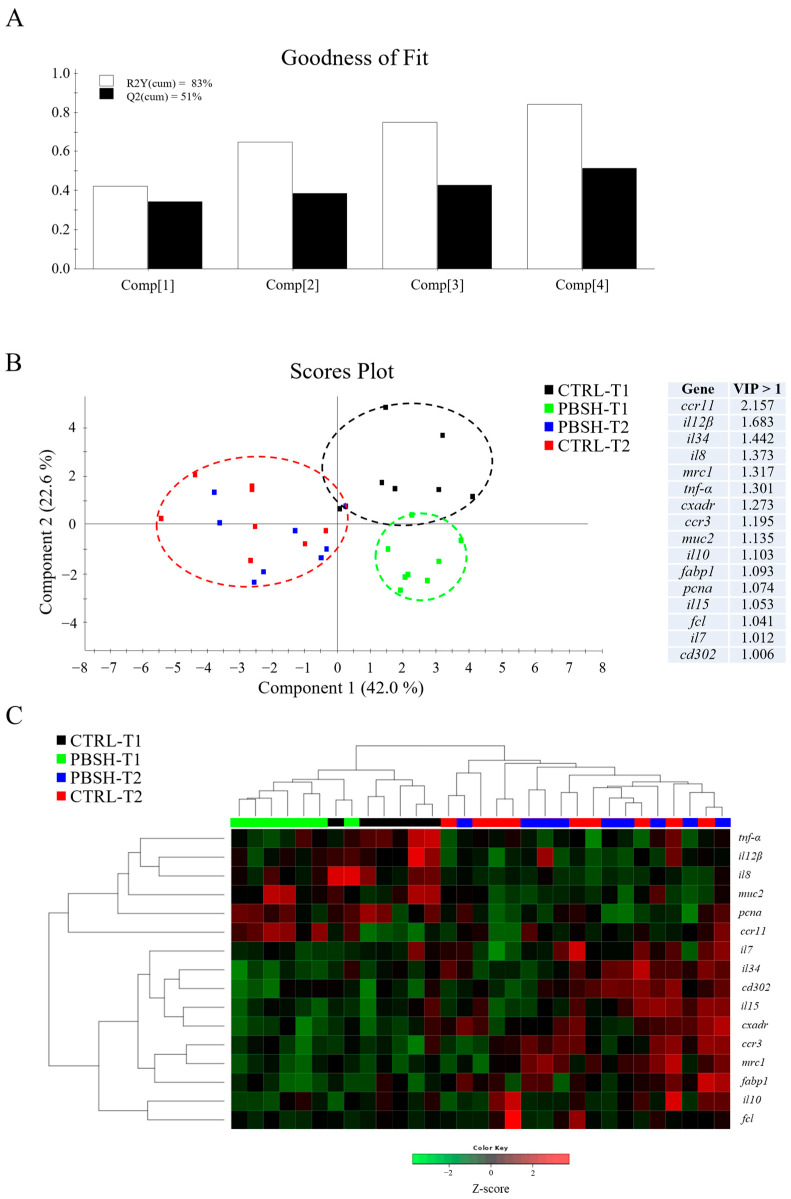
(**A**) Goodness of fit representing the cumulative explained [R2Y(cum), white bars] and predicted [Q2(cum), black bars] variance of each component. (**B**) Two-dimensional partial least-squares discriminant analysis (PLSDA) score plot of anterior intestine data constructed using the two first components in the model. Discriminant genes are ordered by variable importance in the projection (VIP). T1 and T2 refer to samples taken 24 or 48 h post-feeding. (**C**) Heatmap representing the abundance distribution (Z-score) of the genes identified to be driving the separation by diet (VIP ≥ 1).

The multivariate analysis confirmed what had already been described for both liver and intestinal gene expression profiles. In the case of liver, partial least-squares discriminant analysis (PLSDA) accounted for the 86% of variance observed (R2Y) and the 71% of the predicted variance (Q2), whereas for intestine, the PLSDA model exhibited variances of 83% and 51% in R2Y and Q2, respectively (Figure 3A and Figure 4A). For both compartments the results showed a clear separation between dietary groups within the first sampling point, and the union of the two groups (CTRL-T2 and PBSH-T2) belonging to the second point (Figure 3B and Figure 4B). The separation was determined by 22 (liver) and 16 (intestine) genes with a VIP value > 1, confirming the distinct temporal expression profiles. This also highlighted the transient effect of the PBSH diet, which was more pronounced in the liver yet still evident in the intestine, primarily driven by the *ccr11* and *il12β* genes, as shown in the heatmap clustering (Figure 3C and Figure 4C). Validation of both PLSDA models can be found in Figure S1.

### 2.4. Swimming Performance and Fasting/Refeeding Test

**Figure 5:**
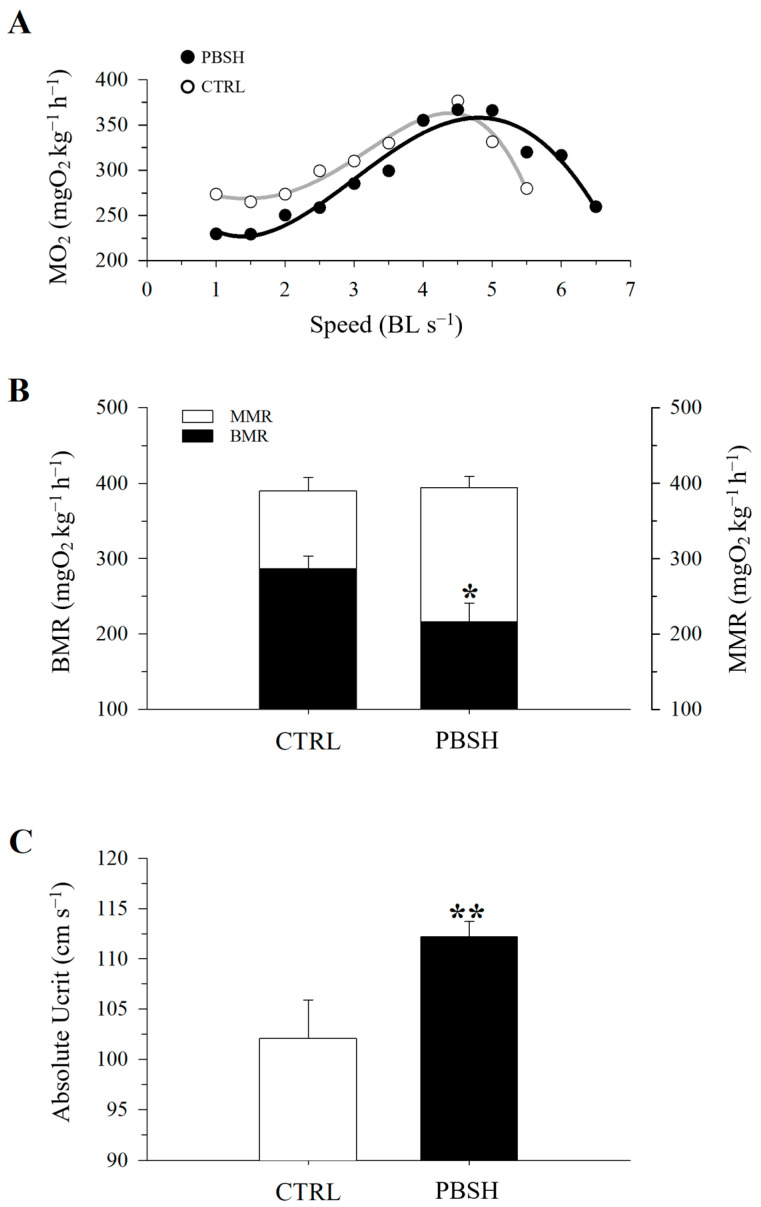
Effect of diet background on swimming performance and measurements of O_2_ consumption in exercised CTRL and PPH (**A**) fish. (**B**) Values of MMR and BMR. (**C**) Values of critical speed. All values are the mean ± SEM of five fish. *, indicate statistically significant differences between groups (Student’s *t*-test, * *p* < 0.1, ** *p* < 0.05).

**Figure 6:**
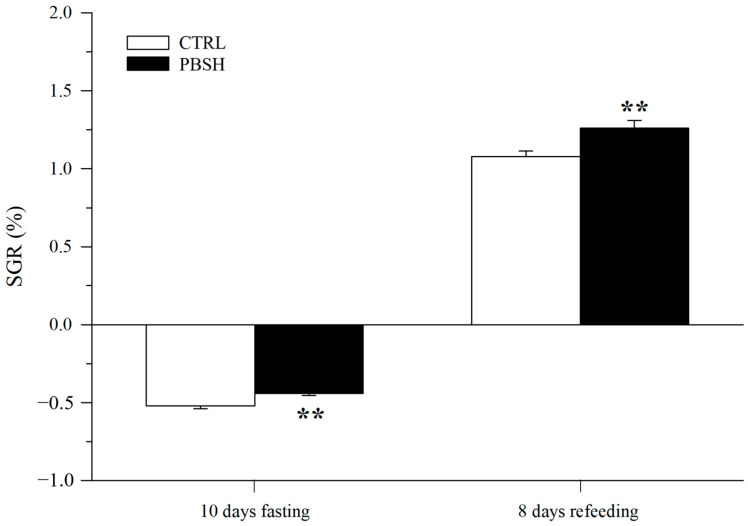
Effect of diet background on growth performance over a subsequent fasting and refeeding period. Values are the mean ± SEM of 24–29 fish from each diet. **, indicate statistically significant differences between experimental groups (Student’s *t*-test, ** *p* < 0.01). SGR = 100 [ln final body weight − ln initial body weight] days^−1^.

Figure 5A reported the effects of the administration of the PBSH diet on swimming performance and the associated O_2_ consumption. The results showed that the fish fed the experimental diet presented significantly lower basal metabolic rates (BMR) at the beginning of the test (216.84 PBSH; 273.52 CTRL) (Figure 5B), and despite reaching the same maximum metabolic rates (MMR), the PBSH group also showed greater resistance to effort, obtaining significantly higher critical speed (Ucrit) values compared to the CTRL, 112.22 and 102.14 cm s^−1^, respectively (Figure 5C). The effect of diets was also tested through a fasting and subsequent re-feeding test. The results achieved also showed that in absence of feed supply, fish fed the PBSH diet exhibited a significantly lower tendency to lose weight compared to the CTRL. Conversely, the opposite trend was achieved during the re-feeding period, in which the experimental group registered a significant higher value of SGR, indicating a greater ability of fish fed diet PBSH to convert food and grow more rapidly during the post-fasting weight regain (Figure 6).

### 2.5. Intestinal Microbiota Diversity and Composition

**Figure 7:**
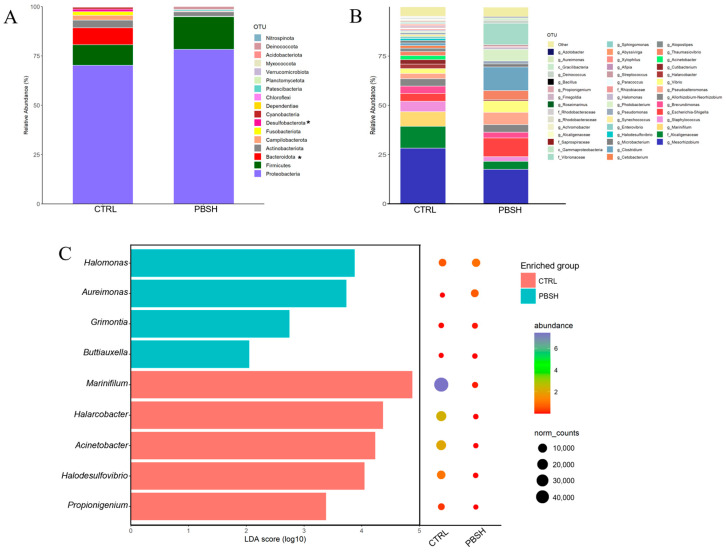
(**A**) Stacked bar chart representing the relative abundance of bacterial phyla of the two experimental diets considering the two temporal (24, 48 h post-feeding) sampling points together (CTRL; PBSH). Superscript asterisks (*) indicate significant differences (Kruskal–Wallis test, *p* < 0.05). (**B**) Stacked bar chart representing the relative abundance of bacterial genera of the two experimental diets (CTRL; PBSH). (**C**) LDA score (log10) of the significant biomarkers and their relative abundances for each diet identified by LEfSe.

The sequencing with ONT produced a total of 702,045 high quality assigned reads for 29 intestinal mucus samples, with an average of 24,208 reads per sample. These reads were assigned to a total of 4683 OTUs and a high percentage of them were classified up to genus level (86.4%), and more than 94% to the level of family, order (96.5%), class (99.4%), and phylum (99.59%). Rarefaction analysis showed curves next to saturation (horizontal asymptote) (Figure S2A) determining adequate coverage of the bacterial community for the number of assigned sequences. The alpha diversity analysis did not highlight significant differences between groups in the richness (Chao1 and ACE) and diversity (Simpson and Shannon) indexes, either considering the effects of diet, sampling times, or their combination (Figure S2B). However, beta-diversity analysis revealed significant differences between the two dietary groups (CTRL; PBSH) regardless of the time (PERMANOVA, F = 1.828, R2 = 0.063, *p* = 0.035), suggesting a higher influence in our study of the diet rather than fasting time (24/48 h post-feeding) on the intestinal microbiota composition. Considering the two sampling points together, microbiota profiles were dominated by Proteobacteria, Firmicutes, and Bacteroidota, which together account for approximately 92% in both groups. Changes at this taxonomic rank were observed for Bacteroidota and Desulfobacterota *phyla*, which registered a significant decrease in the PBSH group, passing from 8.60% to 0.23% and 1.32% to 0.02%, respectively (Figure 7A). At a lower taxonomic level, the most abundant taxa exhibited a slightly different distribution, as almost the total abundance (95%) in the CTRL fish was distributed in 41 OTUs, while in the PBSH group, it was reduced to 22 OTUs (Figure 7B). Within this list, seven OTUs including the genera *Aureimonas*, *Halomonas*, *Marinifilum*, *Halarcobacter*, *Acinetobacter*, *Halodesulfovivrio*, and *Propionigenium*, together with two less abundant ones, were identified as markers for PBSH and/or CTRL diet by the LEfSe analysis (Figure 7C).

## 3. Discussion

To corroborate the in vitro results previously obtained with PBSH [16], its effects as a functional ingredient for fish diet were tested in a feeding trial using gilthead sea bream, applying a multifactorial perspective, which also considered a short to medium temporal scale. For this purpose, a double sampling point, after 24 and 48 h of the end of the feeding trial, together with a larger period of fasting and re-feeding test were carried out. The application of these protocols allowed for the identification of transient, albeit intense, positive effects of the experimental diet with PBSH on fish behavior, stress response, energy management, particularly evident in the liver metabolism and intestinal microbiota populations.

Considering the whole picture, the trial highlighted an optimal growth rate achieved by both dietary groups (CTRL and PBSH), with a daily weight increase of 2.3% and an FCR value of approximately 1. These results are in line with those published by numerous authors which investigated the use of hydrolyzed proteins or porcine meals as a PAP source to replace FM in the fish feeds [17,18,19,20,21,22]. Positive results were also achieved considering the effects of these ingredients in different farmed species such as Atlantic salmon (*Salmo salar*, Linnaeus, 1758), red seabream (*Pagrus major*, Temminck and Schlegel, 1843) and Pacific white shrimp (*Penaeus vannamei*, Boone, 1931) using porcine by-product alone or in combination with other ingredients [8,9,10,23]. In this sense, Gisbert et al. [24] and Resende et al. [25] also described how porcine protein hydrolysate inclusion has the potential to enhance the immune response using an ex vivo assay stimulating gilthead sea bream splenocytes with lipopolysaccharide (LPS) and reducing mortality of European sea bass (*Dicentrarchus labrax*, Linnaeus, 1758) after *Tenacibaculum maritimum* infection, without affecting fish growth. The hepatic and intestinal histology results confirmed this trend, showing a conserved ultrastructure of the organ and no histopathological signs between the two dietary groups for both tissues. The morphological evaluation indeed exhibited a proper distribution of lipids and glycogen within the hepatocytes, and the physiological organization of the mucosal folds, submucosal layers, and muscular layers of the intestine. The beneficial effects of hydrolysates, particularly on the intestine structure, find confirmation in the literature as indicated for red sea bream (*Pagrus major*) and hybrid groupers (*Epinephelus fuscoguttatus* ♀ × *E. lanceolatus* ♂) [26,27].

Going deeper into the cellular and molecular effects of PBSH as functional ingredients, the present study highlighted differences in the typical hematic stress biomarkers. Fish fed CTRL diets exhibited increased levels of cortisol and glucose 48 h after the end of the trial, compared to the first sampling point (only 24 h of fasting). On the contrary, the animals which received the experimental diets did not show variation between the two points, maintaining low levels of both indicators. These data are in line with the in vitro results previously reported, which described the PBSH potential to prolong the half-life of incretin hormones (GLP-1 and GIP), stimulating insulin secretion while suppressing glucagon release in response to glucose loads [16]. Furthermore, these multifunctional peptides can also promote more robust glycemic control, while effectively regulating the appetite and the feed intake of the animals [28]. In intensively reared fish species, feeding time represents a potential source of stress due to aggressive interactions, which can occur before feeding and continue even after its end [29]. Hence, the regulation of the intricate network of central and peripheral signals which control satiety and hunger has a strong influence in social interactions between animals [30,31]. Accordingly, present results highlighted a clear modification in pre-feeding behavior, registering a significant decrease in attacks between specimens and a more relaxed attitude in the 30 min before the daily administration of feed. Apart from better management of the energy sources, less aggressiveness is also related to the higher presence of free amino acids in the PSBS diet that can be easily assimilated by the fish. Numerous studies have demonstrated how different amino acids including tryptophan, phenylalanine, and tyrosine, as hormone precursors and neurotransmitters can modify animals’ behavior and stress response [32,33]. Tryptophan, specifically, due to its contribution in the synthesis of serotonin (5HT), while melatonin and kynurenine compounds can reduce cannibalism and promote an aggression-suppressive effect in several fish species [34,35,36]. These compounds, together with the appetite modulating hormone, have also been shown to have clear effects on the cannabinoid system. As a result, the activation of this complex determines a double consequence, both as an appetite regulator and as a modulator of animal behavior, which ultimately leads to a better use of metabolic energy [37,38].

Plasma glucose levels also represent the first step undertaken by animals to cope with a period of limited food availability [39]. Hence, the lower levels shown by fish fed the PBSH diet also suggest better utilization of dietary sources and greater resistance to fasting and nutriment deprivation, which can lead to a lower catabolic energy expenditure and a consequent reduction in BMR. This theory finds abundant confirmation in the present study. The liver was the organ most affected by the experimental diet both from a biometric and activity point of view. In relation to body weight, the liver size of the PBSH fish showed a significant reduction, indicative of lower hepatic activity [40] and consequently minor lipid deposition in the hepatocytes, which in turn decreases the risk of developing hepatic steatosis. These results were also confirmed at the transcriptomic level, as the gene expression analysis showed a general but transient down-regulation of the hepatic genes directly correlated with the lipogenesis, such as the biosynthesis of long chain FAs (*elovl4*) and triglycerides (*scd1b*), but also with lipid uptake, including *hl* and *lpl*, which determined an overall reorganization of the available energy [41,42,43]. Regarding this latter aspect, mitochondrial respiration showed a similar trend. Numerous genes related with sensing metabolism and beta oxidation, including *ucp1*, *cpt1a*, and *hif1α*, also highlighted a lower level of expression indicating an energy conservation strategy [44]. A drastic decrease in metabolism, along with the switch to a hypometabolic state, represents a common physiological adaptation that fish can use as a compensative strategy to overcome the risk of oxidative stress and ROS over-production due to adverse environmental conditions such as hypoxia, increase stocking density, and temperature [45,46,47,48]. However, differently from these conditions which mostly increase lipogenesis, and a re-adjustment of O_2_ utilization in mitochondrial machinery [44], the present results are more likely to suggest a change to a less energy-consuming status. Basal metabolism in fish is relatively constant under steady environmental conditions; therefore, the differences found in this work would indicate that the nutritional advantages of the experimental diet favor a lower energy investment for catabolic functions and the maintenance of homeostasis. Feed composition is indeed a source of flexibility in BMR [49,50]. In the present study, further confirmation of this assumption comes from the results of the forced swimming test. Since the PBSH fish consume a lower amount of O_2_ at rest, this advantage translates into a higher performance, characterized by a 20% higher critical speed, but the same MMR. Reaching MMR in animal performance represents the shift from aerobic to anaerobic metabolism, determining the reduction in ATP production efficiency and the accumulation of by-products such as lactic acid [47,51,52,53]. Accordingly, the positive effect of the present experimental diet allowed the animals to experience fatigue later, reaching the level of maximum O_2_ consumption at higher speeds, significantly increasing their athletic performance.

This metabolic advantage, exhibited by the fish fed PBSH, became even more evident at the systemic level over the fasting and re-feeding period. The lack of nourishment intake generally forces the mobilization of energy substrates, such as hepatic glycogen reserves as the first metabolic fuel, followed by lipids and proteins and the entrance to a catabolic state, for preserving physiological homeostasis [54,55,56,57]. This state is characterized by low growth rate and substantial weight loss. In line with this, after an initial reduction in SGR exhibited by both groups, fish fed with the PBSH diet showed a lower propensity to lose weight, corroborating what was previously observed at the cellular level and confirms, in the medium–long term, the positive contribution of bioactive peptides and free amino acids of the hydrolysate in the management of body energy, even in fasting conditions. Furthermore, during the 8 days of re-feeding, these same animals exhibited a greater growth capacity, reaching significantly higher values of SGR compared to the CTRL. The return to a normal feeding regimen normally induces a hyper-anabolic status in which animals attempt to accelerate the growth rates to compensate the starvation-induced slowdown partially or totally [58,59]. This tendency has been described in different fish species including sea bream and Nile tilapia (*Oreochromis niloticus*, Linnaeus, 1758) [52,60]. Hence, the differences shown in the present study further highlighted how this functional ingredient maximizes weight recovery and the growth rate. This aspect is particularly important to certify that this dietary solution, among others [32,61], can be especially useful to metabolically prepare the animals for stressful or physiologically “disadvantageous” moments that can occur during the animals’ production cycle, such as those related to climate change.

In the current study, time variation also represents an important driving variable. The results, in fact, highlighted a temporal dynamic which modulates the advantage provided by using the experimental diet. The induced state of basal metabolism, evident in the liver gene expression, exhibited a strong tendency to fade after 48 h post feeding, following a general increasing pattern that ultimately converged to similar values between the CTRL and PBSH group. This trend is not surprising, as the genes affected are consistent with the onset of the physiological response to fasting. The functions involved have indeed to do with processes related to the synthesis and re-absorption of bile acids [62,63], energy balance and oxidative metabolism [64,65], and the use of lipids as secondary fuel source after glycogen reserves [54,55,57,66]. The positive effect provided by the experimental diet was also observed at the intestinal level, although to a lesser extent. The results showed a transient effect in the PBSH group associated with an anti-inflammatory profile, particularly represented by the lower values of *il12β* and *cx32.2*. The first gene represents a promoter of the differentiation of T-helper cells and the cytotoxic action of natural killer cells, while the second has been shown to have an active role in reducing the expression level of LPS-induced pro-inflammatory cytokine IL-8 and TNF-alpha in Japanese flounder (*Paralichthys olivaceus*, Temminck and Schlegel, 1846) [67,68]. Furthermore, *hes1b* also exhibited a significant response from the experimental diet. Although the function of this gene in fish immune response remains less clear, in mammals it restrains inflammation through the modulation of the neutrophil-mediated responses and the production of macrophage-derived chemokines [69]. These results, even if to a lesser degree, show a trend consistent with the in vitro outcomes, which indicated PBSH anti-inflammatory properties associated with the inhibition of TACE and MAGL enzymes [16]. However, in the same way described for the liver, the intestinal sample exhibited a strong temporal dynamic regardless of the diet. This change in the expression profile is mainly due to cytokine and chemokine production and the increase in active immune cells, such as macrophage and leukocytes, which indicate the onset of an inflammatory process. Many studies have confirmed the tendency of the intestine to go through different states of deterioration due to starvation, starting from the alteration of the macro structure, especially affecting the length and width of intestinal villi, mucosal thickness, and the density of the goblet cell population [70,71,72]. In the present results, although the fasting time analyzed was very short, the intestinal gene expression already marks the development of a pro-inflammatory response at an early stage. Impaired digestive and absorptive functions together with an overproduction of ROS, and the consequent antioxidant enzymes, were reported for different fish species, including European sea bass, Nile tilapia (*Oreochromis niloticus*), sea bream, and *Onychostoma macrolepis* (Bleeker, 1871) [73,74,75,76]. The rapid temporal response of the transcriptomic profiles exhibited by both the liver and intestine also emphasized their usefulness as an early responsive proxy to monitor and anticipate the development of processes that could negatively influence the animals’ physiology before they show visible signs.

In addition to the intestinal architecture, adherent microbiota also represents a good indicator of the general health of an organism. Gut bacterial composition presents a high sensitivity to environmental and host-related changes [77,78,79]. However, the reaction times of this diverse group of organisms are highly variable and the mechanisms that regulate it are not entirely clear. In humans, different studies demonstrated that microbiota composition could change in a short temporal scale [80], mainly through drastic interventions, such as antibiotics administration [81,82] and fecal transplant [83,84]; however, in animals, this aspect is still poorly documented. The present results showed that the gut microbiota was not affected by the temporal variable, while they emphasized differences between the two experimental diets. In fact, although no differences in species richness were detected, the diversity indices, in particular Shannon, showed a tendency to decrease in the PBSH group (*p* = 0.08). The influence of the hydrolysate was most evident in the distribution of the top abundant OTUs. In this fraction, apart from more generalist bacteria, the PBSH group also include the genus *Halomonas*, which is generally used as a probiotic in fish diets due to its capacity to produce poly-β-hydroxybutyrate [85,86,87]. Otherwise, our results showed that the experimental diet determined a reduction in the number of bacteria, but at the same time an increase in their relative abundance. Such a trend is comparable to the effect of genetics, in combination with diet, on intestinal microbial community. In fact, both Piazzon et al. [88] and Naya-Català et al. [89] described how selected sea bream families exhibited a more stable and cohesive gut microbiota which at the same time resulted in being more functionally plastic. The changes observed in the present work, even though to a lesser extent, present a strong similarity. In other words, the functional ingredient, PBSH, achieved a modulation of the microbiota, not from a taxonomic point of view, but rather, amplifying the importance of the abundant bacteria. This aspect is important since it demonstrates that these bacteria shared a greater weight within the population both from a hierarchical and therefore functional point of view [90].

## 4. Materials and Methods

### 4.1. Ethics Statement

All procedures including fish husbandry, manipulation, and tissue sampling were approved by the Ethics and Animal Welfare Committee of the Institute of Aquaculture Torre de la Sal (IATS), CSIC Ethics Committee (1295/2022), and Generalitat Valenciana (2022 VSC PEA 0230). They were carried out in the IATS’s registered aquaculture infrastructure facility (code ES120330001055), in accordance with the principles published in the European Animal Directive (2010/63/EU) and Spanish laws (Royal Decree RD53/2013) for the protection of animals used in scientific experiments.

### 4.2. Diets

Two practical diets were formulated and manufactured by Sparos Lda (Portugal) using alternative feed ingredients to meet all the nutrient requirements of fast-growing juveniles of gilthead sea bream (Table S1). Both diets were formulated using the same base feed formulation, with the only exception of the inclusion of porcine blood meal (5%) in the control diet (CTRL) instead of the spray-dried porcine protein hydrolysate at the same inclusion level in the PBSH diet. Details on the methodology used to obtain the PBSH functional ingredient are reported in detail by Moreno-Mariscal (2025) [16]. Briefly, porcine blood was diluted using bidistilled water and then pretreated with ultrasound (1 h, 35 kHz). Subsequently, the compound was enzymatically hydrolyzed in a stirred reactor (Scharlau, Sentmenat, Barcelona, Spain) using Alcalase 4.0 L (2%, 2 h, 65 °C) and Protana™ Prime (5%, 16 h, 55 °C), ultrafiltered with 10 kDa membranes under nitrogen pressure and ultimately spray-dried to obtain the final PBSH ingredient. The choice of PBSH functional ingredient for the gilthead sea bream diet was based on the promising results obtained in vitro, including the high content of essential and functional amino acids, bioactive peptides with antioxidant, anti-inflammatory properties, and enzyme-inhibitory activities (e.g., DPP-IV, NEP, TACE, MGL).

### 4.3. Animals and Feeding Trial

Gilthead sea bream juveniles (mean body weight, 7.5 g) of Mediterranean origin (Avramar, Burriana, Spain) were transported to IATS facilities, where they were acclimated for 2 months in 3000 L tanks. After the acclimation period, fish with an initial body weight (IBW) of approximately 19.5 g, were pit-tagged in the dorsal musculature with passive integrated transponders (ID-100A 1.25 Nano Transponder; Trovan, Madrid, Spain), being then distributed in duplicated 500 L tanks connected to a flow-through system (30 fish per tank; 60 fish per dietary condition). Fish were maintained under the natural photoperiod (≈14:10 light/dark cycle) and temperature (25.11–29.7 °C) conditions at our latitude (40°5′ N; 0°10′ E) over the course of the entire trial. Fish were individually weighted and measured at the beginning and at the end of the trial using a FR-200 FishReader W (Trovan, Madrid, Spain) to evaluate growth indices, including specific growth rates (SGR) and feed conversion ratio (FCR). The feeding trial lasted 10 weeks (July–August 2023), in which fish were fed using automated feeders distributing the feed ration in three meals per day (9:00, 12:00, and 15:00 h) near to visual satiety. During the trial, temperature and water O_2_ concentration (>75% saturation for all the duration of the trial) were continuously measured through an online environmental monitoring system. Weekly determinations of un-ionized ammonia were performed using Orion Dual Star pH, ISE meter and ammonia electrode Orion 9512HPBNWP (Thermo Scientific, Beverly, MA, USA), with levels always below the toxic threshold level (<0.05 mg/L).

### 4.4. Sample Collection Following Feeding Trial

Sample collection took place at the end of the feeding trial in two consecutive events, 24 and 48 h post feeding. For this purpose, a total of 16 fish for each experimental group, equally distributed in the two days of sampling, were anesthetized with 0.1 g/L MS-222 (Sigma, Saint Louis, MO, USA), and then blood samples were taken from caudal vessels using heparinized syringes. Subsequently, blood samples were centrifuged at 3000× *g* for 20 min at 4 °C and stored until processing. The same fish were then euthanized by cervical section and opened to dissect the liver and viscera to calculate the hepatosomatic (HSI) index and viscerosomatic (VSI) index, respectively. For both sampling events, portions of the liver and anterior intestine (AI) were collected and put in RNAlater solution for transcriptomic analysis. The remaining portion of AI (~2 cm) was opened and washed with sterile Hank’s balanced salt solution before collecting the autochthonous intestinal bacteria by scraping the intestinal mucus with the blunt edge of a sterile scalpel. Mucus samples were then transferred to a sterile Eppendorf tube and maintained in ice until subsequent DNA extraction. Samples for histological analysis were only collected in the first sampling event. To this end, liver and portions of AI and posterior intestine (PI) were fixed in 10% neutral buffered formalin (pH = 7.2) and stored at 4 °C until further processing and morpho-pathological evaluations.

### 4.5. Behavioral Monitoring and Swimming Performance Test

After completion of the feeding trial, fish were kept separated in the two experimental groups and fed for one more week, following the same feeding regime. During this time, the pre-feeding behavior was monitored by video recording, starting from 30 min before the daily meals. The videos were obtained using cameras positioned above the tank. In parallel, to monitor the swimming performance and the metabolic activity rate (O_2_ consumption), five fish randomly selected from each condition were subjected to an exercise test using a swim tunnel system (dimensions 10 × 10 × 40 cm, Model PA10500, Loligo^®^ Systems, Viborg, Denmark). The test included measurements of BMR and MMR, that serve to determine how much energy an organism uses while at rest and during peak activity, respectively. The Ucrit, defined as the maximum speed at which fish can swim before exercise exhaustion, was also determined.

### 4.6. Fasting Weight Loss and Re-Feeding Weight Regain

To further evaluate the effect of nutritional background on metabolic rates, the remaining fish per experimental condition (30 fish per diet) were subjected to a fasting phase for ten days and a subsequent re-feeding phase for eight days. Fish weights were measured individually at the beginning of the fasting period, and before/after the re-feeding, to calculate the fasting weight loss and the weight regain during the re-feeding phase.

### 4.7. Biochemical and Gene Expression Analyses

Plasma glucose was determined using the Invitrogen™ Glucose Colorimetric Detection Kit (Invitrogen, EIAGLUC, Carlsbad, CA, USA). Plasma cortisol levels were determined with an enzyme Immunoassay Kit (Arbor Assays, K003-H1W, Ann Arbor, MI, USA) following the manufacturer’s indications. Total plasma cholesterol was determined using a cholesterol esterase/cholesterol dehydrogenase reagent (ThermoFisher Scientific, Middletown, VA, USA). Triglycerides were analyzed using a commercial kit (981786, ThermoFisher Scientific, Vantaa, Finland). For transcriptomic purposes, tissue RNA extraction and analysis followed the same protocol described elsewhere [44]. Briefly, liver and AI samples were extracted using the MagMAX-96 total RNA isolation kit (Life Technologies, Carlsbad, CA, USA) after homogenization in TRI reagent following manufacturers’ instructions. RNA quantity and purity were determined by Nanodrop (Thermo Scientific, Waltham, MA, USA). Reverse transcription of RNA was performed with random decamers using the High-Capacity cDNA Archive Kit (Applied Biosystems, Foster Coty, CA, USA). Real-time quantitative PCR was carried out with an Eppendorf Mastercycler Ep Realplex, using a customized 96-well PCR array layout. Specific PCR primer pair sequences are listed in Supplementary Tables S2 and S3. A total of 101 genes were analyzed, divided into 57 and 44 genes for liver and AI, respectively (Table 2 and Table 3). The genes comprised in the liver array included markers of the Gh/Igf system (9), lipid metabolism (14), transcription factors and nuclear receptors (8), oxidative metabolism and energy sensing (16), and antioxidant defense (10). The genes in the AI array comprised markers of epithelial integrity (11), mucus production (2), nutrient transport (4), cytokines and chemokine-related proteins (13), T cell and monocyte/macrophage markers (4), pattern recognition receptors (8), and immunoglobulins (2). Specific primer pair sequences for liver and AI are listed in Tables S2 and S3, respectively. Controls of general PCR performance were included in each array, and all the pipetting operations were performed by means of an EpMotion 5070 Liquid Handling Robot (Eppendorf, Hamburg, Germany). For multigene expression analysis, all values in each tissue were referenced to the expression of a given gene in CTRL fish with an arbitrary assigned value of 1, namely the peroxisome proliferator-activated receptor α (*pparα*) in liver and the C-C chemokine receptor type 9 (*ccr9*) in AI.

### 4.8. Histological Analysis

Fixed samples of liver, AI, and PI were processed using standard histological procedures and embedded in paraffin. Sections (4 µm thick) were stained with Giemsa and with periodic acid-Schiff (PAS) and examined with a Leitz Dialux 22 light microscope and an Olympus DP70 camera (Leica, Hesse, Germany). Histological alterations were registered according to a semiquantitative scoring scale (from 0 = absence to 3 = very abundant/severe). In intestinal sections, inflammatory markers (i.e., abundance of intraepithelial lymphocytes, abundance of eosinophilic granular cells, degree of submucosal hyperplasia), goblet cell abundance, and degree of lipid vacuolization in enterocytes were quantified. In liver sections, the degree of lipid and glycogen storage in hepatocytes was scored.

### 4.9. Bacterial DNA Extraction, Sequencing and Bioinformatic Analysis

Autochthonous bacterial DNA was extracted from the intestinal mucus samples (200 μL) using the High Pure PCR Template Preparation Kit (Roche, Basel, Switzerland) following the manufacturer’s instructions, including a previous lysis step with lysozyme (Sigma) as described elsewhere [91]. The DNA concentration and quality were checked using NanoDrop 2000c (Thermo Fisher Scientific, Waltham, MA, USA) and agarose gel electrophoresis (1% *w*/*v* Tris-EDTA buffer). Then, the extracted DNA samples were stored at −20 °C until sequencing. Intestinal microbiota composition was obtained according to the methods described by Domingo-Bretón et al. [92]. Briefly, the complete V1-V9 region of the 16s rRNA gene was amplified using 27F (5′-AGAGTTTGATCM TGGCTCAG-3′) and 1492R (5′-CGGTTACCTTGTTACGACTT-3′) primers pairs and the PCR conditions optimized elsewhere [93]. PCR products were purified using Agencourt AMPure XP beads (Backman Coulter, Brea, CA, USA) and then visualized in agarose gel (1% agarose in TAE buffer) and quantified using the Quant-iT PicoGreen dsDNA Assay Kit (Thermo Fisher Scientific, Waltham, MA, USA). Libraries were barcoded using the Native Barcoding kit 96 V14 (SQK-NBD114.96) and sequenced in a MinION (Oxford Nanopore Technologies, Oxford, UK) sequencing device, using a flow-cell R10.4.1 (FLO-MIN114). Sequencing data was acquired using MinKNOW v24.02.6 software. Raw sequencing files were basecalled, demultiplexed, and trimmed from barcodes and adapters using Dorado v0.7. The resulting FASTQ files were uploaded to the Sequence Read Archive (SRA) under the Bioproject accession number PRJNA1263604 (BioSample accession numbers: SAMN48534451-479). Raw reads were filtered using Chopper v.0.8.0 while quality and length metrics were obtained using NanoPlot v1.42.0 [94]. Filtered reads were then taxonomically assigned with Minimap2 v2.28-r1209 (H. Li, 2021), using SILVA v138.1 as a reference database [95].

### 4.10. Statistical Analysis

Data of growth performance, organosomatic indexes, pre-feeding behavior, blood biochemistry, swim performance, and fasting/re-feeding test results were analyzed by Student’s *t*-test using SigmaPlot v15 (Systat Software Inc., San Jose, CA, USA). For all data, normality distribution was verified by Shapiro–Wilk test. Differences in transcriptomic data were analyzed by Student’s *t*-test for comparison between experimental groups within the same sampling point, and by two-way ANOVA, with diet and sampling time as fixed factors. To further investigate the separation between experimental groups, a partial least-squares discriminant analysis (PLS-DA) was performed using EZinfo v3.0 (Umetrics, Umeå, Sweden). Outliers were reported at a Hotelling T2 distance > 0.99 and excluded from the model. The model fitness (R2Y) and prediction ability (Q2) were assessed by a validation test using the ropls R package v1.38.0. The contribution to the groups separation of the different genes was determined by the variable importance in projection (VIP) value. VIP score > 1 was considered the threshold level to determine discriminant variables in the PLS-DA model. Histological scoring was evaluated using the non-parametric Kruskal–Wallis test. For microbiota data, rarefaction curves, species richness estimates, and alpha diversity indices were obtained using phyloseq package for R v1.50.0 [96]. Statistical differences in species richness and alpha diversity indices were determined by Kruskal–Wallis test, with a significance threshold of *p* < 0.05. Beta diversity among groups was determined by permutational multivariate analysis of variance (PERMANOVA), using the non-parametric method adonis from the R package vegan v2.6-8 (https://cran.r-project.org/package=vegan, accessed on 10 September 2025) with 1000 random permutations. Differences in bacterial relative abundances were tested using Kruskal–Wallis test, with a significance threshold of *p* < 0.05. To determine the bacteria genera that most likely explain the differences between experimental groups, a linear discriminant analysis (LDA) effect size (LEfSe) was conducted using R package microbiome Marker v1.9.0 (LDA cutoff = 2, Wilcoxon cutoff = 0.05).

## 5. Conclusions

This study highlights the potential of the PBSH as a promising strategy to enhance the significant nutritional and functional benefits of porcine blood as a functional ingredient for aquaculture feed formulations. Although no differences in growth rates and feed conversion were detected, the PBSH diet significantly modulated the expression of genes involved in energy metabolism and inflammation, leading to better stress and welfare indicators, including reduced cortisol and aggressiveness, enhanced swimming capacity, lower basal metabolism with reduced weight loss during fasting, in association with an improved re-feeding response, and a more plastic intestinal microbiota. Overall, these results highlight the bioactive qualities of PBSH and its central role as a functional ingredient, which highlights both transcriptional transient benefits, and persistent physiological advantages, strengthening the fish’s resilience to cope with potential environmental challenges during the production cycle (high temperatures, low O_2_ availability, high culture density, etc.). These findings support the development of sustainable, health-promoting aquafeeds, also valorizing animal by-products within a circular bioeconomy framework. Ultimately, the use of PBSH as a smart nutritional strategy represents a promising pathway toward a welfare-oriented improvement of aquaculture production.

## 6. Patents

Patent: Mora, L., Moreno, C., Moroni, F., Pérez, J., Toldrá, F, ‘Mix of peptides and its use in feed’, Spain, CSIC. Spanish reference: P202430790, International reference: PCT/ES2025/070588.

## Figures and Tables

**Table 1:** Data on growth performance, organosomatic indexes (24 h post-feeding), and pre-feeding behavior of gilthead sea bream fed CTRL or PBSH diets. Data on growth performance and behavior are the mean ± SEM of duplicate tanks. Data on organosomatic indexes are the mean ± SEM of 16 fish (8 fish/tank). Statistically significant differences are analyzed by Student’s *t*-test.

	CTRL	PBSH	*p* ^1^
Initial body weight (g)	19.45 ± 0.01	19.47 ± 0.08	0.815
Final body weight (g)	105.91 ± 0.43	105.90 ± 0.66	0.991
Initial body length (cm)	9.28 ± 0.04	9.32 ± 0.03	0.419
Final body length (cm)	15.87 ± 0.07	15.88 ± 0.06	0.860
Feed intake (g DM/fish)	86.16 ± 1.08	86.78 ± 0.11	0.621
Initial CFK ^2^	2.43 ± 0.04	2.41 ± 0.02	0.658
Final CFK	2.65 ± 0.05	2.64 ± 0.02	0.895
Viscera weight (g)	6.71 ± 0.21	6.52 ± 0.20	0.505
Liver weight (g)	1.33 ± 0.08	1.25 ± 0.06	0.421
VSI (%) ^3^	6.30 ± 0.20	6.22 ± 0.15	0.740
HSI (%) ^4^	1.34 ± 0.05	1.19 ± 0.05	0.041
SGR (%) ^5^	2.30 ± 0.01	2.29 ± 0.00	0.423
FCR ^6^	1.00 ± 0.01	1.01 ± 0.01	0.293
F_A_^7^	0.18 ± 0.01	0.12 ± 0.02	0.021

^1^ Student’s *t*-test, *p*-value. ^2^ Fulton’s body condition factor, CFK = 100 × (body weight/standard length^3^). ^3^ Viscerosomatic index, VSI = 100 × (viscera weight/fish weight). ^4^ Hepatosomatic index, HSI = 100 × (liver weight/fish weight). ^5^ Specific growth rate, SGR = 100 × (ln final body weight − ln initial body weight)/days. ^6^ Feed conversion ratio, FCR = 100 × (dry feed intake/wet weight gain). ^7^ F_A_, frequency of attacks (counts fish^−1^ h^−1^), 30 min prior to feeding time over 1-week period.

**Table 2:** PCR-array layout for liver gene expression profiling.

Function	Gene	Symbol	GenBank
GH/IGF system	Growth hormone receptor I	*ghr1*	AF438176
	Growth hormone receptor II	*ghr2*	AY573601
	Insulin-like growth factor-I	*igf1*	AY996779
	Insulin-like growth factor-II	*igf2*	AY996778
	Insulin-like growth factor binding protein 1a	*igfbp1a*	KM522771
	Insulin-like growth factor binding protein 1b	*igfbp1b*	MH577189
	Insulin-like growth factor binding protein 2a	*igfbp2a*	MH577190
	Insulin-like growth factor binding protein 2b	*igfbp2b*	AF377998
	Insulin-like growth factor binding protein 4	*igfbp4*	KM658998
			
Lipid metabolism	Fatty acid synthase	*fasn*	JQ277708
	Elongation of very long chain fatty acids 1	*elovl1*	JX975700
	Elongation of very long chain fatty acids 4	*elovl4*	JX975701
	Elongation of very long chain fatty acids 5	*elovl5*	AY660879
	Elongation of very long chain fatty acids 6	*elovl6*	JX975702
	Fatty acid desaturase 2	*fads2*	AY055749
	Stearoyl-CoA desaturase 1a	*scd1a*	JQ277703
	Stearoyl-CoA desaturase 1b	*scd1b*	JQ277704
	Cholesterol 7-alpha-monooxygenase	*cyp7a1*	KX122017
	Adipose triglyceride lipase	*atgl*	JX975711
	Hepatic lipase	*hl*	EU254479
	Lipoprotein lipase	*lpl*	AY495672
	85kDa calcium-independent phospholipase A2	*pla2g6*	JX975708
	Hormone sensitive lipase	*hsl*	EU254478
			
Transcription factors and nuclear receptors	Hepatocyte nuclear factor 4 alpha	*hnf4a*	FJ360721
	Sterol regulatory element-binding proteins 1	*srebp1*	JQ277709
	Sterol regulatory element-binding protein 2	*srebp2*	XM_030408996
	Farnesoid X receptor	*fxr*	XM_030426192
	Liver X receptor α	*lxra*	FJ502320
	Peroxisome proliferator-activated receptor α	*pparα*	AY590299
	Peroxisome proliferator-activated receptor β	*pparβ*	AY590301
	Peroxisomeproliferator-activated receptor γ	*pparγ*	AY590304
			
Oxidative metabolism and energy sensing	Hypoxia inducible factor-1 alpha	*hif1α*	JQ308830
	Carnitine palmitoyltransferase 1A	*cpt1a*	JQ308822
	Fatty acid trasnlocase/CD36	*fat/cd36*	XM_030440140
	Fatty acid binding protein, heart	*h-fabp*	JQ308834
	Citrate synthase	*cs*	JX975229
	NADH-ubiquinone oxidoreductase chain 2	*nd2*	KC217558
	NADH-ubiquinone oxidoreductase chain 5	*nd5*	KC217559
	Cytochrome c oxidase subunit I	*cox1*	KC217652
	Cytochrome c oxidase subunit II	*cox2*	KC217653
	Proliferator-activated receptor gamma coactivator 1 alpha	*pgc1α*	JX975264
	Sirtuin 1	*sirt1*	KF018666
	Sirtuin 2	*sirt2*	KF018667
	Sirtuin 3.1a/Sirtuin 3.1b	*sirt3.1a/b*	OR394775(6)
	Sirtuin 3.2	*sirt3.2*	AHX56275
	Sirtuin 5a	*sirt5a*	AHX56277
	Sirtuin 5b	*sirt5b*	OR394777
			
Antioxidant defense	Uncoupling protein 1	*ucp1*	FJ710211
	Glutathione peroxidase 1	*gpx1*	DQ524992
	Glutathione peroxidase 4	*gpx4*	AM977818
	Peroxiredoxin 3	*prdx3*	GQ252681
	Peroxiredoxin 5	*prdx5*	GQ252683
	Superoxide dismutase [Cu-Zn]	*cu-zn-sod/sod1*	JQ308832
	Superoxide dismutase [Mn]	*mn-sod/sod2*	JQ308833
	Glucose-regulated protein 170 kDa	*grp170*	JQ308821
	Glucose-regulated protein 94 kDa	*grp94*	JQ308820
	Glucose-regulated protein 75 kDa	*grp75*	DQ524993

**Table 3:** PCR array layout for anterior intestine gene expression profiling.

Function	Gene	Symbol	GenBank
Epithelial integrity	Proliferating cell nuclear antigen	*pcna*	KF857335
	Transcription factor HES-1-B	*hes1b*	KF857344
	Krueppel-like factor 4	*klf4*	KF857346
	Claudin-12	*cldn12*	KF861992
	Claudin-15	*cldn15*	KF861993
	Cadherin-1	*cdh1*	KF861995
	Cadherin-17	*cdh17*	KF861996
	Tight junction protein ZO-1	*tjp1*	KF861994
	Desmoplakin	*dsp*	KF861999
	Gap junction Cx32.2 protein	*cx32.2*	KF862000
	Coxsackievirus and adenovirus receptor homolog	*cxadr*	KF861998
		
Mucus production	Mucin 2	*muc2*	JQ277710
	Mucin 13	*muc13*	JQ277713
		
Nutrient transport	Intestinal-type alkaline phosphatase	*alpi*	KF857309
	Liver type fatty acid-binding protein	*fabp1*	KF857311
	Intestinal fatty acid-binding protein	*fabp2*	KF857310
	Ileal fatty acid-binding protein	*fabp6*	KF857312
		
Cytokines and chemokine-related proteins	Tumor necrosis factor alpha	*tnfα*	AJ413189
	Interleukin-1 beta	*il1β*	AJ419178
	Interleukin-6	*il6*	EU244588
	Interleukin-7	*il7*	JX976618
	Interleukin-8	*il8*	JX976619
	Interleukin-10	*il10*	JX976621
	Interleukin-12 subunit beta	*il12* *β*	JX976624
	Interleukin-15	*il15*	JX976625
	Interleukin-34	*il34*	JX976629
	C-C chemokine receptor type 3	*ccr3*	KF857317
	C-C chemokine receptor type 9	*ccr9*	KF857318
	C-C chemokine receptor type 11	*ccr11*	KF857319
	C-C chemokine CK8/C-C motif chemokine 20	*ck8/ccl20*	GU181393
		
T cell and monocyte/macrophage markers	Cluster of differentiation 4	*cd4-1*	AM489485
	Cluster of differentiation 8 beta	*cd8b*	KX231275
	Macrophage colony-stimulating factor 1 receptor 1	*csf1r1*	AM050293
	Macrophage mannose receptor 1	*mrc1*	KF857326
		
Pattern recognition receptors (PRRs)	Galectin-1	*lgals1*	KF862003
	Galectin-8	*lgals8*	KF862004
	Toll-like receptor 2	*tlr2*	KF857323
	Toll-like receptor 5	*tlr5*	KF857324
	Toll-like receptor 9	*tlr9*	AY751797
	CD209 antigen-like protein D	*cd209d*	KF857327
	CD302 antigen	*cd302*	KF857328
	Fucolectin	*fcl*	KF857331
		
Immunoglobulins	Immunoglobulin M	*igm*	JQ811851
	Immunoglobulin T membrane-bound form	*igt-m*	KX599201

## Data Availability

All the basecalled microbiota data (FASTQ files) used in this work were loaded in the Sequence Read Archive (SRA) under the Bioproject accession number PRJNA1263604 (BioSample accession numbers: SAMN48534451-479). The rest of the data presented are reported within the article.

## Supplementary Materials

The following supporting information can be downloaded at: https://www.mdpi.com/article/10.3390/ijms262110725/s1.

## Abbreviations

The following abbreviations are used in this manuscript:

PBSH	Spray-dried Porcine Blood Hydrolysate
FM	Fish Meal
FO	Fish Oil
ABPs	Animal By-Products.
PAP	Processed Animal Proteins
FAAs	Free Amino Acids
CTRL	Control Diet
IBW	Initial Body Weight
SGR	Specific Growth Rates
FCR	Feed Conversion Ratio
HIS	Hepatosomatic Index
VSI	Viscerosomatic Index
AI	Anterior Intestine
PI	Posterior Intestine
BMR	Basal Metabolic Rate
MMR	Maximal Metabolic Rate
Ucrit	Critical Speed
PAS	Periodic Acid-Schiff
OTUs	Operational Taxonomic Units
