# Supplementary material for: Feeding for Well-Being: Porcine Blood Hydrolysate Supplementation Improves Metabolic and Welfare-Related Traits in Farmed Gilthead Sea Bream (Sparus aurata)
Source: Int J Mol Sci. 2025 Nov 4;26(21):10725. doi: 10.3390/ijms262110725 (PMC12609438; doi:10.3390/ijms262110725)
Supplement: Supplementary file 1 [file ijms-26-10725-s001.zip › ijms-3897382-supplementary.pdf]

Supplementary

Feeding for Well-being: Porcine Blood Hydrolysate Supplementation Improves Metabolic and Welfare-Related Traits in Farmed Gilthead Sea Bream (*Sparus aurata*)

Cristina Moreno-Mariscal [1], Paul Holhorea [2], Federico Moroni [2,*], Leticia Mora [1*], Fidel Toldrá [1] and Jaume Pérez-Sánchez [2]

Table S1. Ingredients and proximate composition of experimental diets.

Ingredient (%)	CTRL	PPH
**Fishmeal 60**	5.00	5.00
Fish protein hydrolysate	3.00	3.00
Poultry meal	12.00	12.00
Porcine blood meal	**5.00**	0.00
Porcine protein hydrolysate (PPH)	0.00	**5.00**
Feathermeal hydrolysate	5.00	5.00
PROTE-IN HP55	11.10	11.10
Aminopro NT70 – C. glutamicum	6.00	6.00
Corn gluten meal	7.20	7.20
Soybean meal 44	9.00	9.00
Sunflower meal 40	5.00	5.00
Wheat meal	8.88	8.88
Whole peas	5.00	5.00
Wheat starch	2.00	2.00
Vitamin and mineral premix[1]	1.00	1.00
Vitamin E50	0.10	0.10
Antioxidant	0.20	0.20
Sodium propionate	0.10	0.10
MAP (Monoamonium phosphate)	1.65	1.65
L-Lysine HCl 99%	0.30	0.30
Yttrium oxide	0.02	0.02
Fish oil	5.40	5.40
Algae oil	0.90	0.90
Rapeseed oil	6.15	6.15
Proximate composition		
Dry matter (DM, %)	97.10	97.10
Crude protein (% DM)	47.0	47.10
Crude fat (% DM)	16.0	16.0
Crude fiber (% DM)	3.9	3.9
EPA+DHA (% DM)	2.14	2.14

[1] Vitamin and mineral premix: Vitamins (IU or mg/kg diet): DL-alpha tocopherol acetate, 1084.5 mg; sodium menadione bisulphate, 31.3 mg; retinyl acetate, 26,846 IU; DL-cholecalciferol, 4,153 IU; thiamine, 37.5 mg; riboflavin, 37.7 mg; pyridoxine, 40 mg; cyanocobalamin, 0.3 mg; nicotinic acid, 500.9 mg; folic acid, 18.6 mg; ascorbic acid, 625.7 mg; inositol, 619 mg; biotin, 1.9 mg; calcium pantothenate, 125.4 mg; choline chloride, 138.9 mg; betaine, 1238.7 mg. Minerals (g or mg/kg diet): Na, 3 g; Mg, 1 g; K, 5 g; Cu, 13 mg; Fe, 265 mg; I, 3.5 mg; Mn, 26.9 mg; Se, 0.6 mg; Zn, 105.9 mg.

Table S2. Primers for qPCR amplification of hepatic genes.

Gene	Symbol	GenBank	Primer
Growth hormone receptor I	*ghr1*	AF438176	F: ACC TGT CAG CCA CCA CAT GA<br>R: TCG TGC AGA TCT GGG TCG TA
Growth hormone receptor II	*ghr2*	AY573601	F: GAG TGA ACC CGG CCT GAC AG<br>R: GCG GTG GTA TCT GAT TCA TGG T
Insulin-like growth factor-I	*igf1*	AY996779	F: TGT CTA GCG CTC TTT CCT TTC A<br>R: AGA GGG TGT GGC TAC AGG AGA TAC
Insulin-like growth factor-II	*igf2*	AY996778	F: TGG GAT CGT AGA GGA GTG TTG T<br>R: CTG TAG AGA GGT GGC CGA CA
Insulin-like growth factor binding protein 1a	*igfbp1a*	KM522771	F: ACA AAC CAA AAC AGT GCG AGT CCT C<br>R: CCG TTC CAA GAG TTC ACA CAC CAG
Insulin-like growth factor binding protein 1b	*igfbp1b*	MH577189	F: GCC AAA CAG TGT GAG TCA TC<br>R: ATC TTC TTC CCG TTC CAG G
Insulin-like growth factor binding protein 2a	*igfbp2a*	MH577190	F: CCA GCA AAG AGA CCA CCT<br>R: TCT TCA TCT CCT GCC TGT G
Insulin-like growth factor binding protein 2b	*igfbp2b*	AF377998	F: AGC GAT GTG TCC TGA GAT AGT GAG<br>R: GCA CCG TGG CGT GTA GAC C
Insulin-like growth factor binding protein 4	*igfbp4*	KM658998	F: GGC ATC AAA CAC CCG CAC AC<br>R: ATC CAC GCA CCA GCA CTT CC
Fatty acid synthase	*fasn*	JQ277708	F: ACA GGC AGC GTC GGT GCC AGT GGT CTA C<br>R: TCC AGG ACG CAG CCT GCC GCG AAC TAC
Elongation of very long chain fatty acids 1	*elovl1*	JX975700	F: CTT CCT ACA CAT CTT CCA CCA CTC<br>R: CCA TTC CAC CAG GAG CAA AGG
Elongation of very long chain fatty acids 4	*elovl4*	JX975701	F: CGG TGG CAA TCA TCT TCC<br>R: TCA ACT GGC TGT CTG TGT
Elongation of very long chain fatty acids 5	*elovl5*	AY660879	F: CCT CCT GGT GCT CT ACA AT<br>R: GTG AGT GTC CTG GCA GTA
Elongation of very long chain fatty acids 6	*elovl6*	JX975702	F: GTG CTG CTC TAC TCC TGG TA<br>R: ACG GCA TGG ACC AAG TAG T
Fatty acid desaturase 2	*fads2*	AY055749	F: GCA GGC GGA GAG CGA CGG TCT GTT CC<br>R: AGC AGG ATG TGA CCC AGG TGG AGG CAG AAG
Stearoyl-CoA desaturase 1a	*scd1a*	JQ277703	F: CGG AGG CGG AGG CGT TGG AGA AGA AG<br>R: AGG GAG ACG GCG TAC AGG GCA CCT ATA TG
Stearoyl-CoA desaturase 1b	*scd1b*	JQ277704	F: GCT CAA TCT CAC CAC CGC CTT CAT AG<br>R: GCT GCC GTC GCC CGT TCT CTG
Cholesterol 7-alpha-monooxygenase	*cyp7a1*	KX122017	F: CCC TGC TAT TAA AGT CCC ACC TCT<br>R: ATC GTA GGT AGG CTG GAG GAT TC

Adipose triglyceride lipase	*atgl*	JX975711	F: GTG CTT CAG TCC TGG ATG TCT TC<br>R: AGC CTT GCA GGT CCA TGT TGA
Hepatic lipase	*hl*	EU254479	F: TTG TAG AAG GTG AGG AAA ACT G<br>R: GCT CTC CAT CAG ACC ATC C
Lipoprotein lipase	*lpl*	AY495672	F: CGT TGC CAA GTT TGT GAC CTG<br>R: AGG GTG TTC TGG TTG TCT GC
85kDa calcium-independent phospholipase A2	*pla2g6*	JX975708	F: CGC CAA GGA ACT CGG AAA GAT GCT<br>R: ACC GCA CAG CCA TCA GAG TCT
Hormone sensitive lipase	*hsl*	EU254478	F: GCT TTG CTT CAG TTT ACC ACC ATT TC<br>R: GAT GTA GCG ACC CTT CTG GAT GAT GTG
Hepatocyte nuclear factor 4 alpha	*hnf4a*	FJ360721	F: TCG GAG GTT CTG CCA ATG AG<br>R: TGC TGA GGT GCT CCT GAA C
Sterol regulatory element-binding proteins 1	*srebp1*	JQ277709	F: AGG GCT GAC CAC AAC GTC TCC TCT CC<br>R: GCT GTA CGT GGG ATG TGA TGG TTT GGG
Sterol regulatory element-binding protein 2	*srebp2*	XM_030408996	F: TCA GCA GGC TCT ACA CAG AAC<br>R: GAA CGG CAG GTC GTG AGA
Farnesoid X receptor	*fxr*	XM_030426192	F: TAC TTC GCT CGT CTC CTG<br>R: CAT CTC TGC GTG GTA GTG
Liver X receptor α	*lxra*	FJ502320	F: GCA CTT CGC CTC CAG GAC AAG<br>R: CAG TCT TCA CAC AGC CAC ATC AGG
Peroxisome proliferator-activated receptor α	*pparα*	AY590299	F: TCT CTT CAG CCC ACC ATC CC<br>R: ATC CCA GCG TGT CGT CTC C
Peroxisome proliferator-activated receptor β	*pparβ*	AY590301	F: AGG CGA GGG AGA GTG AGG ATG AGG AG<br>R: CTG TTC TGA AAG CGA GGG TGA CGA TGT TTG
Peroxisomeproliferator-activated receptor γ	*pparγ*	AY590304	F: CGC CGT GGA CCT GTC AGA GC<br>R: GGA ATG GAT GGA GGA GGA GGA GAT GG
Hypoxia inducible factor-1 alpha	*hif1α*	JQ308830	F: CAG ATG AGC CTC TAA CTT GTG GAC<br>R: TTA GCA AGA ATG GTG GCA AGA TGA G
Carnitine palmitoyltransferase 1A	*cpt1a*	JQ308822	F: GTG CCT TCG TTC GTT CCA TGA TC<br>R: TGA TGC TTA TCT GCT GCC TGT TTG
Fatty acid trasnlocase/CD36	*fat/cd36*	XM_030440140	F: AGG CGA GGG AGA GTG AGG ATG AGG AG<br>R: CTG TTC TGA AAG CGA GGG TGA CGA TGT TTG
Fatty acid binding protein, heart	*h-fabp*	JQ308834	F: CTG GGT GTG GGC TTC GCT AC<br>R: CTC TGT GTT CTT GAT GGT GCT CTG
Citrate synthase	*cs*	JX975229	F: TCC AGG AGG TGA CGA GCC<br>R: GTG ACC AGC AGC CAG AAG AG
NADH-ubiquinone oxidoreductase chain 2	*nd2*	KC217558	F: TAG GTT GAA TGA CCA TCG TA<br>R: GGC TAA GGA GTT GAG GTT
NADH-ubiquinone oxidoreductase chain 5	*nd5*	KC217559	F: CCT AAA CGC CTG AGC CCT GG<br>R: GCT GTA AAC GAG GTG GCT AGA AGG

Cytochrome c oxidase subunit I	*cox1*	KC217652	F: GTC CTA CTT CTT CTG TCC CTT CCT GTT CT<br>R: AGG TTT CGG TCT GTA AGG AGC ATT GTA ATC
Cytochrome c oxidase subunit II	*cox2*	KC217653	F: ACT GCC TAC ACA GGA CCT TGC C<br>R: GTC TGC TTC CAG GAG ACG GAA TTG T
Proliferator-activated receptor gamma coactivator 1 alpha	*pgc1α*	JX975264	F: CGT GGG ACA GGT GTA ACC AGG ACT C<br>R: ACC AAC CAA GGC AGC ACA CTC TAA TTC T
Sirtuin 1	*sirt1*	KF018666	F: GGT TCC TAC AGT TTC ATC CAG CAG CAC ATC<br>R: CCT CAG AAT GGT CCT CGG ATC GGT CTC
Sirtuin 2	*sirt2*	KF018667	F: GAA CAA TCC GAC GAC AGC AGT GAA G<br>R: AGG TTA CGC AGG AAG TCC ATC TCT
Sirtuin 3.1a/Sirt 3.1b	*sirt3.1a/b*	OR394775(6)	F: CGG AGG AAC TAC AAG GGA GAG GAG<br>R: GTC CCG CTC ATC ACA TCT GGT CG
Sirtuin 3.2	*sirt3.2*	AHX56275	F: CTG CCA AGT CCT CAT CCC<br>R: CTT CAC CAG ACG AGC CAC
Sirtuin 5a	*sirt5a*	KF018670	F: CAG ACA TCC TAA CCC GAG CAG AG<br>R: CCA CGA GGC AGA GGT CAC A
Sirtuin 5b	*sirt5b*	OR394777	F: CGG TCT ACG GAT GTG CTC CAC TAA TGC<br>R: GTC TTG TTG TGT CCA CTA CAG GTC CTC TCA
Uncoupling protein 1	*ucp1*	FJ710211	F: GCA CAC TAC CCA ACA TCA CAA G<br>R: CGC CGA ACG CAG AAA CAA AG
Glutathione peroxidase 1	*gpx1*	DQ524992	F: GAA GGT GGA TGT GAA TGG AAA AGA TG<br>R: CTG ACG GGA CTC CAA ATG ATG G
Glutathione peroxidase 4	*gpx4*	AM977818	F: TGC GTC TGA TAG GGT CCA CTG TC<br>R: GTC TGC CAG TCC TCT GTC GG
Peroxiredoxin 3	*prdx3*	GQ252681	F: ATC AAC ACC CCA CGC AAG ACT G<br>R: ACC GTT TGG ATC AAT GAG GAA CAG ACC
Peroxiredoxin 5	*prdx5*	GQ252683	F: GAG CAC GGA ACA GAT GGC AAG G<br>R: TCC ACA TTG ATC TTC TTC ACG ACT CC
Superoxide dismutase [Cu-Zn]	*cu-zn-sod / sod1*	JQ308832	F: TCA CGG ACA AGA TGC TCA CTC TC<br>R: GGT TCT GCC AAT GAT GGA CAA GG
Superoxide dismutase [Mn]	*mn-sod / sod2*	JQ308833	F: CCT GAC CTG ACC TAC GAC TAT GG<br>R: AGT GCC TCC TGA TAT TTC TCC TCT G
Glucose-regulated protein, 170 kDa	*grp170*	JQ308821	F: CAG AGG AGG CAG ACA GCA AGA C<br>R: TTC TCA GAC TCA GCA TTT CCA GAT TTC
Glucose-regulated protein, 94 kDa	*grp94*	JQ308820	F: AAG GCA CAG GCT TAC CAG ACA G<br>R: CTT CAG CAT CAT CGC CGA CTT TC
Glucose-regulated protein, 75 kDa	*grp75*	DQ524993	F: TCC GGT GTG GAT CTG ACC AAA GAC<br>R: TGT TTA GGC CCA GAA GCA TCC ATG

Beta-actin	*actb*	KY388508	F: TCC TGC GGA ATC CAT GAG A<br>R: GAC GTC GCA CTT CAT GAT GCT

Table S3. Primers for qPCR amplification of anterior intestine genes.

Gene	Symbol	GenBank	Primer
Proliferating cell nuclear antigen	*pcna*	KF857335	F: CGT ATC TGC CGT GAC CTG T<br>R: AGA ACT TGA CTC CGT CCT TGG
Transcription factor HES-1-B	*hes1b*	KF857344	F: GCC TGC CGA TAT GAT GGA A<br>R: GGA GTT GTG TTC ATG CTT GC
Krueppel-like factor 4	*klf4*	KF857346	F: ACA TCA CCG CAC GCA CAC<br>R: AAC CAC AGC CCT CCC AGT C
Claudin-12	*cldn12*	KF861992	F: CTC TCA GGG CTA CAC ATC TAC CTA TGC<br>R: ACA TTC GTG AGC GGC TGG AG
Claudin-15	*cldn15*	KF861993	F: CCG ATT GTG GAA GTA GTG GCT CTG GT<br>R: CAG CAT CAC CCA ACC GAC GAA CC
Cadherin-1	*cdh1*	KF861995	F: TGC TCC ATA CAG CGT CAC CTT ACA<br>R: CTC GTT CAT CCT AGC CGT CCA GTT
Cadherin-17	*cdh17*	KF861996	F: GAT GCC CGC AAC CCA GAG<br>R: CCG TTG ATT CAC TGC CGT AGA C
Tight junction protein ZO-1	*tjp1*	KF861994	F: AAG CAG TAT TAC GGT GAC TCA<br>R: TGC ATC CCT GGC TTG TAG
Desmoplakin	*dsp*	KF861999	F: GCA GAA GGA GCA CGA GAC CATC<br>R: GGG TGT TCT TGT CGC AGG TGA A
Gap junction Cx32.2 protein	*cx32.2*	KF862000	F: CGA GGT GTT CTA TCT GCT CTG TA<br>R: CTT GTG GGT GCG AGT CCT
Coxsackievirus and adenovirus receptor homolog	*cxadr*	KF861998	F: CAT CAG AGG ACT ACG AGA GG<br>R: CAT CTT GGC AGC ATT TGG T
Mucin 2	*muc2*	JQ277710	F: ACG CTT CAG CAA TCG CAC CAT<br>R: CCA CAA CCA CAC TCC TCC ACA T
Mucin 13	*muc13*	JQ277713	F: TTC AAA CCC GTG TGG TCC AG<br>R: GCA CAA GCA GAC ATA GTT CGG ATA T
Intestinal-type alkaline phosphatase	*alpi*	KF857309	F: CCG CTA TGA GTT GGA CCG TGA T<br>R: GCT TTC TCC ACC ATC TCA GTA AGG G
Liver type fatty acid-binding protein	*fabp1*	KF857311	F: GTC CTC GTC AAC ACC TTC ACC AT<br>R: CGC CTT CAT CTT CTC GCC AGT
Intestinal fatty acid-binding protein	*fabp2*	KF857310	F: CGA GCA CAT TCC GCA CCA AAG<br>R: CCC ACG CAC CCG AGA CTT C
Ileal fatty acid-binding protein	*fabp6*	KF857312	F: ACC CAG GAC GGC AAT ACC<br>R: CGA CGG TGA AGT TGT TGG T
Tumor necrosis factor alpha	*tnfα*	AJ413189	F: CAG GCG TCG TTC AGA GTC TC<br>R: CTG TGG CTG AGA GGT GTG AG

Interleukin-1 beta	*il1β*	AJ419178	F: GCG ACC TAC CTG CCA CCT ACA CC<br>R: TCG TCC ACC GCC TCC AGA TGC
Interleukin-6	*il6*	EU244588	F: TCT TGA AGG TGG TGC TGG AAG TG<br>R: AAG GAC AAT CTG CTG GAA GTG AGG
Interleukin-7	*il7*	JX976618	F: CTA TCT CTG TCC CTG TCC TGT GA<br>R: TGC GGA TGG TTG CCT TGT AAT
Interleukin-8	*il8*	JX976619	F: CAG CAG AGT CTT CAT CGT CAC TAT TG<br>R: AGG CTC GCT TCA CTG ATG G
Interleukin-10	*il10*	JX976621	F: AAC ATC CTG GGC TTC TAT CTG<br>R: GTG TCC TCC GTC TCA TCT G
Interleukin-12 subunit beta	*il12β*	JX976624	F: ATT CCC TGT GTG GTG GCT GCT<br>R: GCT GGC ATC CTG GCA CTG AAT
Interleukin-15	*il15*	JX976625	F: GAG ACC AGC GAG CGA AAG GCA TCC<br>R: GCC AGA ACA GGT TAC AGG TTG ACA GGA A
Interleukin-34	*il34*	JX976629	F: TCT GTC TGC CTG CTG GTA G<br>R: ATG CTG GCT GGT GTC TGG
C-C chemokine receptor type 3	*ccr3*	KF857317	F: CTA CAT CAG CAT CAC CAT ACG CAT CCT<br>R: TGG CAC GGC ACT TCT CCT TCA
C-C chemokine receptor type 9	*ccr9*	KF857318	F: TCC CTG AGT TAA TCT TCG CCC AAG TG<br>R: TGT TGT ATT CGT TGT TCC AGT AGA CCA GAG
C-C chemokine receptor type 11	*ccr11*	KF857319	F: GCT ACG ATT ACA GTT ATG AA<br>R: TAG ATG ATT GGG AGG AAG
C-C chemokine CK8 / C-C motif chemokine 20	*ck8 / ccl20*	GU181393	F: CCG TCC TCA TCT GCT TCA TAC T<br>R: GCT CTG CCG TTG ATG GAA C
Cluster of differentiation 4	*cd4-1*	AM489485	F: TCC TCC TCC TCG TCC TCG TT<br>R: GGT GTC TCA TCT TCC GCT GTC T
Cluster of differentiation 8*β*	*cd8β*	KX231275	F: CCG AAA TGT GGA AGA CTG GAA CTC<br>R: CTT TGG AGG TAA GGT TGG AGG GAT
Macrophage colony-stimulating factor 1 receptor 1	*csf1r1*	AM050293	F: TTG CGT GTG GTG AGG AAG GAA GGT<br>R: AGC AGG CAG GGC AGC AGG TA
Macrophage mannose receptor 1	*mrc1*	KF857326	F: CTT CCG ACC GTA CCT GTA CCT ACT CA<br>R: CGA TTC CAG CCT TCC GCA CAC TTA
Galectin-1	*lgals1*	KF862003	F: GTG TGA GGA GGT CCG TGA TG<br>R: ACT GTA GAG CCG TCC GAT AGG

Galectin-8	*lgals8*	KF862004	F: GGC GGT GAA CGG CGG TCA<br>R: GCT CCA GCT CCA GTC TGT GTT GAT AC
Toll-like receptor 2	*tlr2*	KF857323	F: CAT CTG CGA CTC TCC TCT CTT CCT<br>R: ATT CAA CAA TGG AGC GGT GGA CTT
Toll-like receptor 5	*tlr5*	KF857324	F: TCG CCA ATC TGA CGG ACC TGA G<br>R: CAG AAC GCC GAT GTG GTT GTA AGA C
Toll-like receptor 9	*tlr9*	AY751797	F: GCC TTC CTT GTC TGC TCT TTC T<br>R: GCC GTA GAG GTG CTT CAG TAG
CD209 antigen-like protein D	*cd209d*	KF857327	F: CGC CAC GAG CAT GAG GAC AA<br>R: TCT TGC CAG AAT CCA TCA CCA TCC A
CD302 antigen	*cd302*	KF857328	F: GGA CCA GAG GAA GAG CAC ATC<br>R: GAC CAG GGC GGA CAT CAG
Fucolectin	*fcl*	KF857331	F: CCA TAC TGC TGA ACA GAC CAA CC<br>R: TGA TGG AGG TGA CGA TGT AGG A
Immunoglobulin M	*igm*	JQ811851	F: ACC TCA GCG TCC TTC AGT GTT TAT GAT GCC<br>R: CAG CGT CGT CGT CAA CAA GCC AAG C
Immunoglobulin T membrane-bound form	*igtm*	KX599201	F: AGA CGA TGC CAG TGA AGA GGA TGA GT<br>R: CGA AGG AGG AGG CTG TGG ACC A
Beta-actin	*actb*	KY388508	F: TCC TGC GGA ATC CAT GAG A<br>R: GAC GTC GCA CTT CAT GAT GCT

Table S4. Relative gene expression of liver mRNA transcripts at 24 h and 48 h post-feeding. Values are the mean ± SEM of 7-8 fish. All data are in reference to the expression level of *pparα* in CTRL fish at each sampling time with an arbitrary value of 1. Differentially expressed genes by diet and/or sampling time are in bold.

	24 h post-feeding		Student's t-test	48 h post-feeding		Student's t-test	Two-way ANOVA		
	CTRL	PPH	*p*	CTRL	PPH	*p*	Sampling time	Diet	*Time x Diet*
ghr1	1.38±0.22	0.99±0.20	0.215	1.01±0.16	1.09±0.15	0.724	0.460	0.415	0.219
ghr2	0.95±0.15	0.69±0.11	0.175	1.12±0.14	1.11±0.19	0.987	0.058	0.382	0.396
igf1	4.76±0.57	3.41±0.57	0.117	5.66±0.58	5.65±0.59	0.999	**0.011**	0.252	0.253
igf2	1.35±0.29	0.52±0.06	**0.011**	1.74±0.26	1.12±0.16	0.064	**0.025**	**0.002**	0.626
igfbp1a	0.02±0.01	0.01±0.00	0.364	0.01±0.00	0.02±0.01	0.417	0.920	0.849	0.217
igfbp1b	0.19±0.05	1.45±0.37	**0.007**	0.37±0.08	1.04±0.14	**0.002**	0.612	**<0.001**	0.207
igfbp2a	0.66±0.11	0.45±0.09	0.174	1.05±0.24	1.04±0.19	0.997	**0.007**	0.544	0.549
igfbp2b	1.08±0.15	0.82±0.19	0.291	1.18±0.13	1.11±0.18	0.732	0.244	0.304	0.570
igfbp4	0.53±0.05	0.37±0.04	**0.026**	0.53±0.04	0.59±0.06	**0.449**	**0.028**	0.273	**0.032**
fasn	0.04±0.01	0.03±0.01	0.171	0.03±0.01	0.03±0.00	0.785	0.193	0.211	0.366
elovl1	4.72±0.33	3.53±0.39	**0.035**	4.79±0.50	4.48±0.39	0.628	0.219	0.075	0.290
elovl4	0.23±0.05	0.14±0.02	0.075	0.16±0.01	0.16±0.02	0.889	0.303	0.099	0.079
elovl5	3.17±0.54	2.26±0.14	0.120	1.07±0.17	2.15±0.31	**0.009**	**0.004**	0.811	**0.008**
elovl6	0.40±0.05	0.27±0.05	0.102	0.36±0.07	0.37±0.06	0.948	0.555	0.317	0.271
fads2	0.69±0.16	0.66±0.22	0.900	0.24±0.05	0.36±0.11	0.321	**0.024**	0.781	0.620
scd1a	0.10±0.02	0.06±0.01	0.054	0.07±0.01	0.10±0.02	0.248	0.789	0.709	**0.032**
scd1b	0.56±0.05	0.16±0.03	**<0.001**	0.04±0.01	0.04±0.00	0.512	**<0.001**	**<0.001**	**<0.001**
cyp7a1	0.81±0.16	0.50±0.11	0.134	1.15±0.16	1.03±0.15	0.591	**0.005**	0.152	0.518
atgl	0.05±0.01	0.05±0.01	0.803	0.05±0.01	0.06±0.02	0.351	0.686	0.378	0.580
hl	4.72±0.20	3.16±0.40	**0.004**	4.31±0.41	4.44±0.27	0.801	0.200	**0.040**	**0.017**
lpl	1.36±0.17	0.84±0.07	**0.011**	1.94±0.22	1.70±0.14	0.379	**<0.001**	**0.025**	0.395
pla2g6	0.08±0.02	0.05±0.01	0.246	0.05±0.01	0.06±0.01	0.567	0.358	0.352	0.192
hsl	0.12±0.01	0.11±0.01	0.748	0.15±0.02	0.18±0.01	0.118	**<0.001**	0.371	0.177
hnf4a	0.85±0.09	0.71±0.11	0.321	0.95±0.17	0.87±0.13	0.719	0.303	0.395	0.809
srebp1	0.33±0.04	0.28±0.04	0.426	0.18±0.02	0.21±0.03	0.302	**0.002**	0.916	0.222
srebp2	0.30±0.11	0.21±0.03	0.457	0.16±0.03	0.24±0.04	0.151	0.423	0.945	0.207
fxr	0.30±0.04	0.33±0.04	0.543	0.26±0.03	0.37±0.06	0.102	0.958	0.093	0.356
lxra	0.56±0.08	0.60±0.07	0.711	0.60±0.09	0.59±0.07	0.967	0.865	0.819	0.772
pparα	1.02±0.08	0.83±0.09	0.133	1.11±0.09	1.10±0.08	0.940	**0.042**	0.242	0.286
pparβ	0.14±0.03	0.22±0.02	0.064	0.15±0.02	0.22±0.03	0.059	0.761	**0.008**	0.936
pparγ	0.31±0.03	0.21±0.02	**0.010**	0.27±0.02	0.28±0.02	0.726	0.391	0.054	**0.019**
hif1α	0.47±0.03	0.34±0.03	**0.009**	0.41±0.03	0.45±0.02	0.215	0.382	0.114	**0.003**
cpt1a	0.22±0.03	0.14±0.01	**0.026**	0.36±0.04	0.34±0.03	0.678	**<0.001**	0.095	0.315
fat/cd36	0.01±0.00	0.01±0.00	0.878	0.02±0.00	0.02±0.00	0.233	**<0.001**	0.221	0.267
h-fabp	18.49±2.32	11.89±1.55	**0.033**	12.42±1.53	15.30±1.61	0.219	0.469	0.317	**0.015**
cs	0.45±0.04	0.30±0.03	**0.011**	0.52±0.05	0.44±0.02	0.175	**0.014**	**0.006**	0.413
nd2	13.16±1.45	12.42±2.08	0.777	13.54±1.03	12.71±0.89	0.552	0.820	0.592	0.973
nd5	5.59±0.76	4.54±0.71	0.332	5.33±0.39	4.24±0.38	0.066	0.635	0.080	0.973
cox1	51.90±6.30	36.22±7.83	0.141	53.60±5.12	46.64±5.11	0.352	0.336	0.078	0.487
cox2	22.18±2.24	18.97±4.86	0.557	23.52±1.77	19.94±1.91	0.191	0.701	0.263	0.952
pgc1α	0.05±0.01	0.04±0.01	0.372	0.08±0.02	0.09±0.01	0.767	**0.006**	0.806	0.456
sirt1	0.05±0.00	0.03±0.00	**0.002**	0.05±0.00	0.05±0.00	0.924	**0.002**	0.053	0.074
sirt2	0.11±0.01	0.08±0.01	**0.039**	0.11±0.01	0.11±0.01	0.838	0.054	0.081	0.138
sirt3.1a/b	0.05±0.01	0.04±0.00	0.089	0.05±0.00	0.05±0.00	0.393	**0.017**	0.407	0.059
sirt3.2	0.01±0.00	0.01±0.00	0.652	0.02±0.00	0.02±0.00	0.105	**0.002**	0.468	0.158
sirt5a	0.13±0.01	0.09±0.01	**0.005**	0.14±0.01	0.13±0.01	0.360	**0.014**	**0.004**	0.070
sirt5b	0.06±0.01	0.05±0.01	0.355	0.09±0.01	0.10±0.01	0.556	**<0.001**	0.931	0.300
ucp1	9.04±0.87	6.15±0.70	**0.021**	13.44±1.70	15.32±0.85	0.341	**<0.001**	0.648	**0.039**
gpx1	0.98±0.12	0.62±0.05	**0.013**	1.28±0.16	1.02±0.06	0.146	**0.003**	**0.007**	0.684
gpx4	4.81±0.77	3.39±0.58	0.160	2.93±0.30	3.29±0.32	0.432	0.071	0.328	0.106
prdx3	0.59±0.05	0.41±0.05	**0.030**	0.70±0.06	0.62±0.05	0.323	**0.007**	**0.025**	0.417
prdx5	0.42±0.06	0.21±0.02	**0.005**	0.43±0.04	0.37±0.03	0.315	**0.047**	**0.003**	0.064
cu-zn-sod / sod1	4.34±0.30	2.80±0.30	**0.003**	3.41±0.31	3.10±0.19	0.404	0.257	**0.002**	**0.035**
mn-sod / sod2	0.59±0.05	0.41±0.05	**0.016**	0.67±0.06	0.60±0.03	0.273	**0.009**	**0.011**	0.302
grp170	0.52±0.12	0.35±0.05	0.197	0.59±0.10	0.50±0.08	0.460	0.234	0.146	0.672
grp94	1.83±0.28	1.03±0.15	**0.020**	1.74±0.24	1.59±0.25	0.679	0.310	0.050	0.167
grp75	0.38±0.04	0.22±0.02	**0.001**	0.46±0.05	0.37±0.03	0.101	**0.002**	**<0.001**	0.282

Table S5. Relative gene expression of anterior intestine mRNA transcripts at 24 h and 48 h post-feeding. Values are the mean ± SEM of 7-8 fish. All data are in reference to the expression level of *ccr9* in CTRL fish at each sampling time with an arbitrary value of 1. Differentially expressed genes by diet and/or sampling time are in bold.

	24 h post-feeding		Student's t-test	48 h post-feeding		Student's t-test	Two-way ANOVA		
	CTRL	PPH	*p*	CTRL	PPH	*p*	Sampling time	Diet	*Time x Diet*
pcna	3.33±0.51	3.30±0.28	0.947	2.39±0.25	2.35±0.39	0.943	**0.014**	0.922	0.997
hes1b	1.96±0.34	1.54±0.22	0.329	2.50±0.37	1.54±0.27	0.063	0.390	**0.038**	0.396
klf4	0.76±0.23	0.94±0.18	0.541	0.76±0.21	0.80±0.20	0.901	0.730	0.600	0.726
cldn12	0.40±0.06	0.39±0.05	0.875	0.42±0.05	0.44±0.07	0.831	0.592	0.953	0.791
cldn15	25.59±3.66	24.45±2.44	0.794	26.77±4.32	23.01±3.12	0.492	0.971	0.484	0.708
cdh1	8.34±1.10	8.1±0.79	0.831	7.10±0.81	7.53±1.04	0.749	0.354	0.941	0.704
cdh17	36.84±6.80	31.29±4.05	0.482	30.96±3.95	33.13±4.25	0.714	0.675	0.725	0.425
tjp1	0.22±0.04	0.22±0.03	0.938	0.25±0.05	0.24±0.04	0.810	0.475	0.887	0.812
dsp	3.92±0.26	4.15±0.27	0.563	4.99±0.44	4.50±0.38	0.411	0.051	0.705	0.318
cx32.2	87.45±10.86	75.11±6.83	0.341	82.42±5.49	64.05±4.23	**0.019**	0.269	**0.040**	0.675
cxadr	2.87±0.14	2.75±0.16	0.613	3.40±0.16	3.44±0.19	0.878	**0.001**	0.828	0.653
alpi	79.78±9.06	64.29±3.46	0.116	76.77±14.98	72.00±8.89	0.788	0.817	0.323	0.599
fabp1	90.24±7.17	79.63±5.27	0.247	109.13±8.42	104.58±8.87	0.715	**0.008**	0.328	0.694
fabp2	486.62±86.32	490.24±71.17	0.974	568.70±92.90	545.46±123.77	0.880	0.480	0.918	0.889
fabp6	0.04±0.02	0.02±0.01	0.240	0.07±0.05	0.04±0.03	0.583	0.362	0.303	0.996
muc2	60.32±8.06	59.92±6.71	0.970	39.59±2.72	44.23±5.59	0.448	**0.005**	0.721	0.672
muc13	33.23±1.87	31.95±1.49	0.597	35.16±3.63	36.79±2.03	0.701	0.177	0.944	0.555
tnfα	0.19±0.02	0.16±0.01	0.114	0.14±0.01	0.15±0.01	0.685	0.051	0.283	0.120
il1β	0.10±0.01	0.08±0.01	0.335	0.09±0.01	0.09±0.01	0.678	0.921	0.301	0.607
il6	0.01±0.00	0.02±0.01	0.169	0.01±0.00	0.02±0.01	0.064	0.348	**0.022**	0.366
il7	0.37±0.03	0.32±0.01	0.099	0.43±0.06	0.41±0.04	0.748	0.074	0.311	0.653
il8	0.69±0.10	0.56±0.10	0.364	0.31±0.02	0.38±0.03	0.137	**<0.001**	0.661	0.187
il10	0.11±0.01	0.1±0.01	0.512	0.16±0.02	0.11±0.01	0.067	0.050	**0.049**	0.172
il12β	0.56±0.05	0.43±0.03	**0.030**	0.39±0.04	0.36±0.02	0.542	**<0.001**	**0.031**	0.152
il15	0.62±0.03	0.59±0.02	0.416	0.68±0.05	0.69±0.04	0.914	**0.029**	0.717	0.589
il34	0.60±0.02	0.54±0.03	0.130	0.74±0.04	0.71±0.03	0.653	**<0.001**	0.207	0.586
cd4-1	0.26±0.03	0.32±0.04	0.277	0.38±0.03	0.36±0.05	0.757	**0.032**	0.602	0.330
cd8b	0.06±0.01	0.06±0.01	0.537	0.09±0.02	0.09±0.01	0.823	**0.020**	0.879	0.601
ccr3	0.62±0.07	0.58±0.04	0.615	0.84±0.07	0.82±0.08	0.853	**0.002**	0.651	0.874
ccr9	1.08±0.08	1.07±0.09	0.988	1.45±0.14	1.30±0.09	0.365	**0.009**	0.463	0.474

ccr11	2.42±0.16	3.77±0.15	**<0.001**	2.73±0.18	3.15±0.18	0.126	0.379	**<0.001**	**0.011**
ck8/ccl20	4.44±0.63	4.20±0.98	0.842	6.05±0.84	4.94±0.67	0.327	0.169	0.423	0.608
csf1r1	0.17±0.02	0.17±0.03	0.905	0.22±0.02	0.22±0.02	0.958	**0.047**	0.897	0.948
igm	20.79±9.77	9.14±1.99	0.265	13.59±5.08	7.02±1.10	0.258	0.413	0.117	0.654
igt-m	0.55±0.09	0.81±0.13	0.130	1.34±0.25	0.71±0.13	0.051	0.056	0.286	**0.015**
lgals1	12.37±1.47	13.84±1.30	0.467	14.53±1.83	13.43±0.75	0.591	0.536	0.894	0.367
lgals8	2.84±0.09	2.68±0.20	0.496	2.55±0.14	2.67±0.25	0.671	0.410	0.926	0.449
tlr2	0.53±0.05	0.57±0.05	0.618	0.73±0.11	0.68±0.12	0.738	0.093	0.913	0.620
tlr5	0.03±0.00	0.03±0.00	0.113	0.04±0.00	0.03±0.00	0.288	0.141	0.065	0.854
tlr9	0.04±0.01	0.04±0.00	0.654	0.04±0.01	0.03±0.00	0.485	0.544	0.407	0.815
cd209d	0.12±0.01	0.11±0.01	0.531	0.13±0.01	0.13±0.01	0.871	0.325	0.803	0.612
cd302	8.15±0.43	7.93±0.37	0.701	9.11±0.49	9.25±0.39	0.829	**0.012**	0.924	0.675
mrc1	1.10±0.11	0.89±0.05	0.100	1.29±0.11	1.35±0.09	0.697	**0.002**	0.418	0.165
fcl	4.99±1.52	2.75±1.07	0.243	18.26±8.11	7.39±1.90	0.188	**0.048**	0.141	0.327

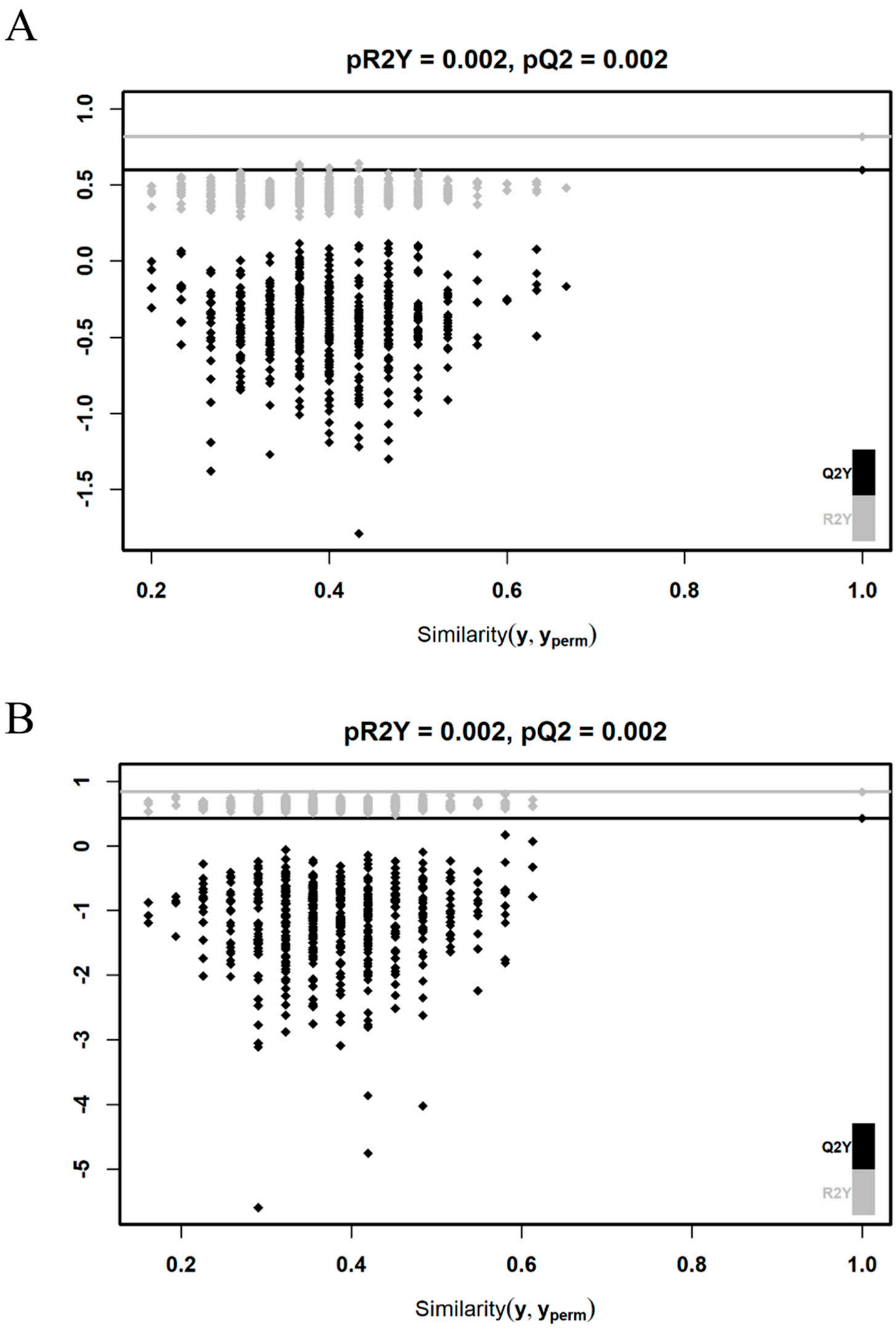

Figure S1. Validation plots.

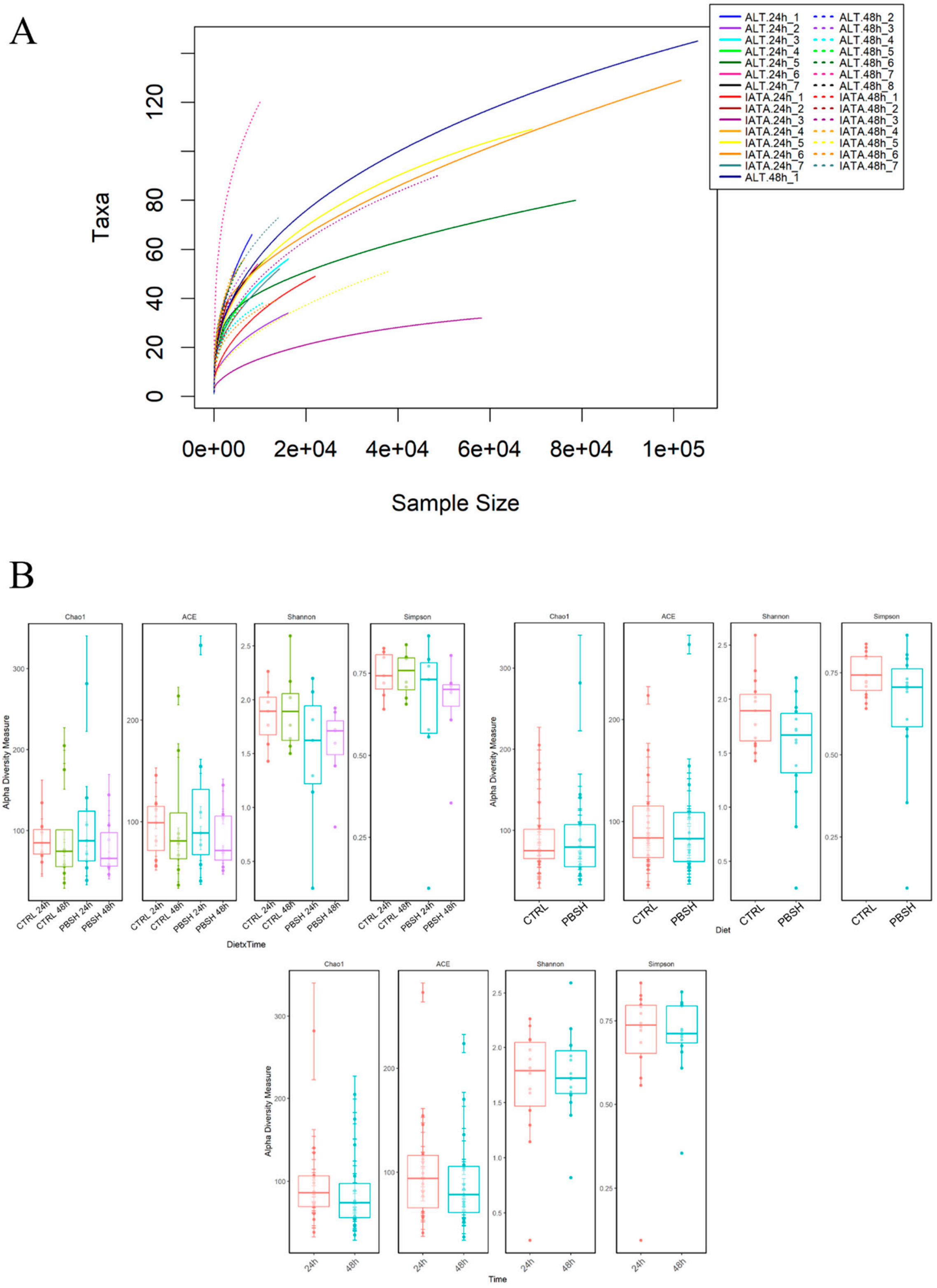

Figure S2. Rarefaction curves and alpha-diversity.
